# Weight loss in MASLD restores the balance of liver fatty acid sources

**DOI:** 10.1172/JCI174233

**Published:** 2025-05-01

**Authors:** Jennifer E. Lambert, Maria A. Ramos-Roman, Maressa J. Valdez, Jeffrey D. Browning, Thomas Rogers, Elizabeth J. Parks

**Affiliations:** 1 Center for Human Nutrition,; 2 Department of Internal Medicine,; 3 Department of Clinical Nutrition, and; 4 Department of Pathology, University of Texas Southwestern Medical Center, Dallas, Texas, USA.

**Keywords:** Hepatology, Metabolism, Clinical practice, Obesity

## Abstract

**BACKGROUND:**

Lipogenesis contributes substantially to the pathological accumulation of intrahepatic triacylglycerol (IHTG) in metabolic dysfunction–associated steatotic liver disease (MASLD). Since hepatic lipogenesis is highly sensitive to energy intake, we hypothesized that mechanisms of MASLD regression induced by weight loss would be driven by a marked reduction in the lipogenic pathway.

**METHODS:**

Overweight adults with high liver fat (HighLF; *n* = 9; IHTG ≥ 5.6% measured by ^1^H-magnetic resonance spectroscopy) or low (normal) liver fat (LowLF; *n* = 6; IHTG < 5.6%) received dietary counseling for 6 months and underwent comprehensive metabolic phenotyping during inpatient studies that captured fasting and fed states. Multiple stable isotopes were used to assess the contribution of lipogenesis, free fatty acids (FFAs), and dietary fat to IHTG.

**RESULTS:**

Body weight loss (–10% ± 2%) reduced IHTG in individuals with MASLD (19.4% ± 3.6% to 4.5% ± 2.1%, *P* < 0.001). Insulin sensitivity improved significantly (46%, *P* < 0.01), while fasting FFA flux from adipose tissue was not different. VLDL-triacylglycerol (VLDL-TG) concentrations fell by 38% (*P* = 0.02) because of a 67% reduction in contribution from lipogenesis (*P* = 0.02), whereas the absolute contributions from FFAs and dietary fat to VLDL-TG were not different. Reduced lipogenesis was significantly associated with loss of IHTG.

**CONCLUSION:**

These data underscore the primary role of lipogenesis in MASLD pathology and highlight the importance of controlling this pathway through treatment strategies.

**TRIAL REGISTRATION:**

ClinicalTrials.gov (NCT01371396).

**FUNDING:**

National Institutes of Health (NIH) grant RL1DK081187; Task Force for Obesity Research at Southwestern (TORS) NIH UL1DE019584; and Clinical and Translational Science Award NIH/National Center for Advancing Translational Sciences UL1-RR024982.

## Abstract 2

This study used specialized techniques to measure changes in liver fatty acid sources and whole-body metabolism leading to resolution of fatty liver after weight loss

## Graphical abstract

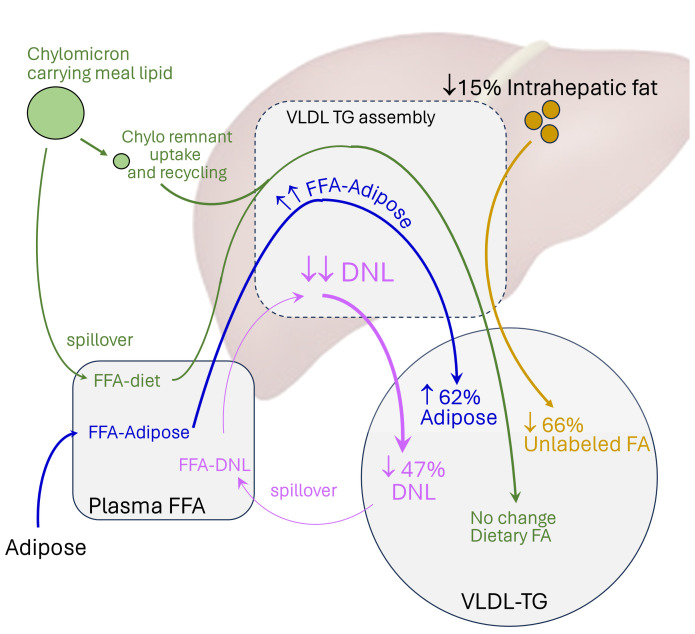

## Introduction

The prevalence of metabolic dysfunction–associated steatotic liver disease (MASLD) is rising in tandem with increasing rates of obesity and insulin resistance in the population (1–4). Over the next 10 years, MASLD will become the most common liver disease in developed countries (5–7). Sources of fatty acids used for intrahepatic triacylglycerol (IHTG) synthesis include plasma free (non-esterified) fatty acids (FFAs) derived from adipose lipolysis, dietary fat, and fatty acids made in the liver via de novo lipogenesis (DNL). Tracer studies using PET imaging have estimated that the liver takes up a constant proportion (~20%–25%) of the fatty acids that flow to the organ (8). Using labeling techniques, unrestrained FFA flux from adipose tissue to the liver was shown to provide the primary source of fatty acids to IHTG, and therefore adipose insulin resistance in MASLD likely contributes to excess IHTG (9, 10). However, we and others (11–13) have shown that DNL is also markedly elevated in individuals with MASLD, even when compared with individuals without MASLD but with a similar degree of obesity and insulin resistance (11). Further, we have shown that DNL is increased in a graded fashion as histologically assessed liver disease worsens (14), that treatment with inhibitors of lipogenesis lowers IHTG (15, 16), and that this reduction can occur in as little as 10 days (17).

To investigate mechanisms of lipid accrual in fatty liver, most studies have focused on the measurement of one of the 3 fatty acid sources (adipose, diet, or DNL). Unfortunately, this strategy does not support discovery of the processes controlling hepatocellular VLDL-triacylglycerol (VLDL-TG) assembly. During maturation of VLDL through the endoplasmic reticulum and Golgi, TGs are added to the particle that can be derived from any of the 3 lipid sources, or from TGs stored in cellular droplets (18). Using multiple isotopes, we have shown that fatty acids from different sources are processed differently in the liver (e.g., a portion of FFAs are immediately re-secreted in VLDL, while de novo fatty acids appear to enter a liver storage pool before secretion, etc.) and that the fatty acid sources contributing to stored IHTG are reflected in the fractional sources found in VLDL-TG out in the plasma (12, 14).

Currently, weight loss remains the safest and most effective treatment for MASLD (19). Weight loss interventions have focused on individuals with obesity and/or type 2 diabetes and achieved impressive reductions in IHTG. For example, IHTG levels can be reduced from 6% at baseline to 2% after 6 weeks of weight loss, or from 12% at baseline to 2% after 7–16 weeks (20, 21). Although it is well established that weight loss can improve liver health (as reviewed by Balakrishnan et al., ref. 22), few studies have quantitated the metabolic mechanisms responsible for resolution of liver fat via weight loss or comprehensively characterized changes in lipid metabolism during this process.

In past studies of lipoprotein fatty acid sources, investigators have compared characteristics of subjects with high IHTG and low IHTG, but the low-IHTG comparison groups had lower or no insulin resistance or were younger and leaner. Our own past data from those with low IHTG were derived from relatively lean subjects (23, 24). In the elegant study of Klein and colleagues, 6 individuals without obesity who lost 10% body weight demonstrated a 35% reduction in DNL present in lipoprotein-TG, and a 50% reduction in IHTG (25). However, without labeling of the diet, it is unknown how intrahepatic lipid processing of all fatty acid sources was altered by weight loss. Here, we sought to compare groups with widely varying IHTG levels while matching for the level of peripheral insulin sensitivity, age, body weight, and blood lipids. DNL is a relatively flexible biochemical pathway, correlates with the magnitude of IHTG (25), is highly responsive to energy/carbohydrate restriction, and is reduced with improvement of insulin sensitivity (26–29). Therefore, we hypothesized that a weight loss program designed to resolve fatty liver would do so primarily by reducing hepatic DNL. Our goal was to understand (a) how these reductions would impact the other sources of liver-TG, and (b) whether the mechanisms would be similar for subjects with low and high IHTG. With equivalent baseline levels of insulin sensitivity in the 2 groups, the results of the intervention would more directly reflect the isolated effect of energy restriction without the confounding effect of different levels of peripheral insulin resistance influencing the results. We found that weight loss–induced alterations in hepatic lipid utilization resulted in similar lowering of DNL and greater hepatic FFA use for TG synthesis, with a greater magnitude of changes observed in the high-IHTG subjects. The absolute use of non-DNL sources was not related to lowering of IHTG. Hypertriacylglycerolemia was improved primarily through reductions in DNL and improved lipoprotein clearance. Given the strong effect of energy restriction to reduce fatty acid synthesis, the results support a principal role of reduced DNL to lead to greater utilization of plasma FFAs for VLDL-TG synthesis and secretion, further leading to reductions in liver fat. These results underscore the pathological, yet highly modifiable, role of DNL in MASLD development.

## Results

**Figure 1:**
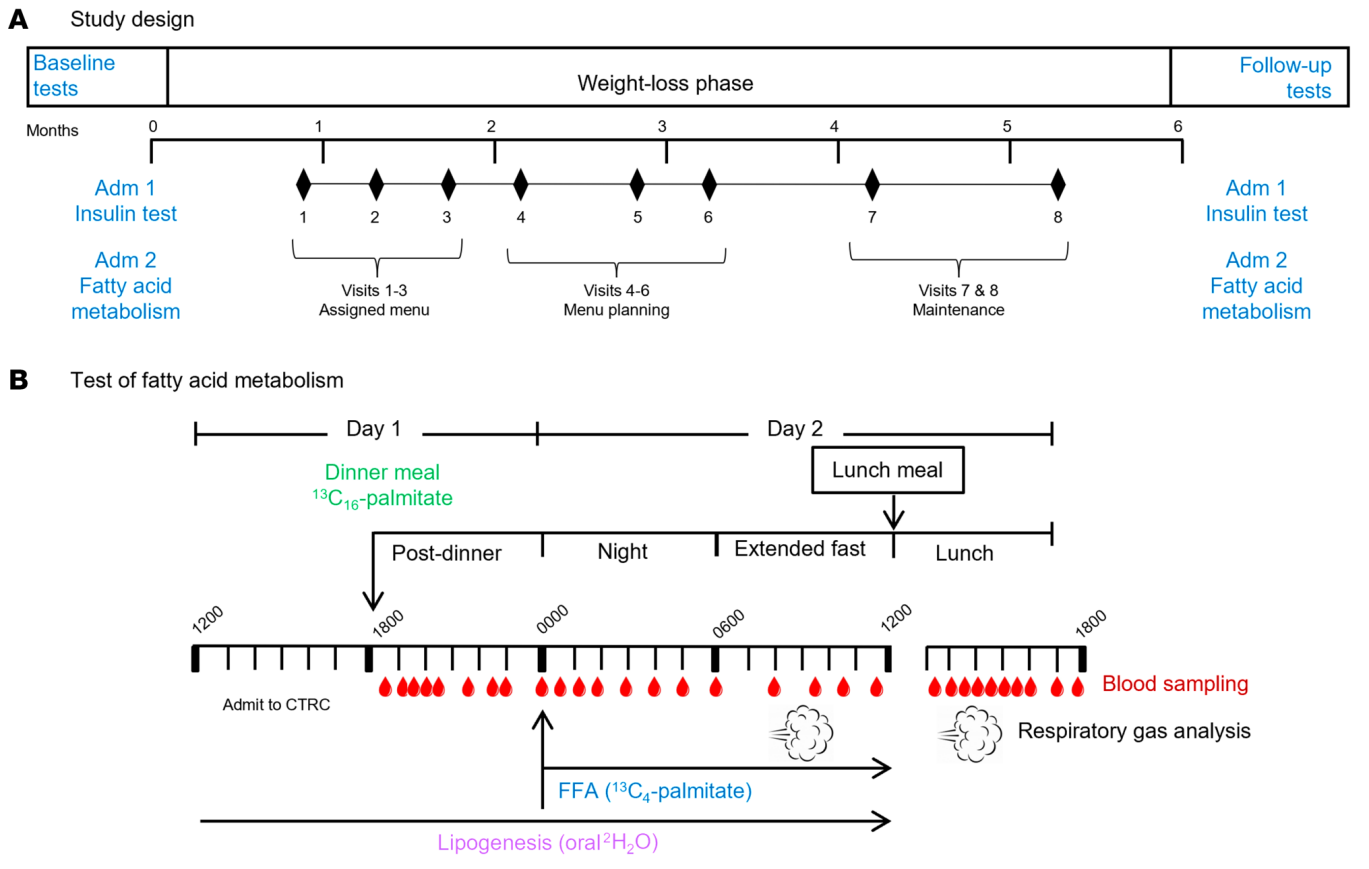
Six-month study design and inpatient protocol to quantitate fatty acid and TG metabolism. (**A**) Subjects were recruited to participate in a 6-month dietary study to determine the metabolic mechanisms of weight loss–induced improvements in liver fat, including insulin metabolism (assessed in admission 1 via a frequently sampled, insulin-modified i.v. glucose tolerance test) and fatty acid metabolism. (**B**) Before and after weight loss, fatty acid metabolism was measured during an overnight isotope infusion study. Oral dosing with deuterated water occurred for 10 days before the inpatient study, and fatty acid isotopes were incorporated into the evening meal and infused i.v. to track adipose FFA flux and the sources of VLDL-TG palmitate. Data from the 24-hour study were analyzed to assess post-meal lipid metabolism, nighttime flux of lipids, and how these processes changed when the subjects fasted an additional 4 hours, followed by a meal. CTRC, Clinical and Translational Research Center; FFA, free fatty acids.

In a previous publication (11), we analyzed metabolic variables in 24 non-diabetic subjects with metabolic syndrome, elevated liver enzymes, and suspected MASLD, who possessed either low liver fat (<5.6%; LowLF) or high liver fat (>5.6%; HighLF) (see recruitment flow in Supplemental Figure 1; supplemental material available online with this article; https://doi.org/10.1172/JCI174233DS1). In that analysis, subjects were matched for insulin resistance, and a distinctive characteristic of those with HighLF was a 2-fold greater hepatic DNL (11). After those baseline studies, 16 of the subjects agreed to enter a 6-month weight loss intervention; of those, 15 completed the intervention and were included for analysis (1 subject was excluded because of poor compliance). The aim of the present study (Figure 1A) was to produce weight loss by reducing overall energy intake, achieved through improvement of diet quality by reduction of intake of simple sugars and increasing consumption of whole foods. By bringing about meaningful weight loss, our goal was to test the influence of energy restriction on fatty acid flux and sources of VLDL-TG fatty acids (primary endpoints), to identify metabolic mechanisms associated with reductions in both IHTG and VLDL-TG (secondary endpoints), and to compare weight loss effects in individuals who started with either high or low liver fat (Figure 1B; secondary endpoints). Admission 1 was a frequently sampled i.v. glucose tolerance test, and admission 2 was designed to comprehensively assess fatty acid metabolism (Figure 1B). By design, the groups differed in their baseline content of IHTG (LowLF 2.1% ± 0.9%, *n* = 6, vs. HighLF 19.4% ± 1.2%, *n* = 9, *P* < 0.001), but were matched for the results of the i.v. glucose tolerance tests, age (45 ± 6 years and 50 ± 9 years, *P* = 0.292), body weight, dietary intakes, anthropometrics, and fasting biochemistries (Table 1). However, insulin concentrations (*P* = 0.058) and static markers of insulin resistance (homeostatic model assessment for insulin resistance [HOMA-IR] and adipose insulin resistance [AdipoIR], an index of adipose insulin sensitivity) still tended to be higher in the HighLF (Table 2).

### Effect of weight loss on liver fat.

**Figure 2:**
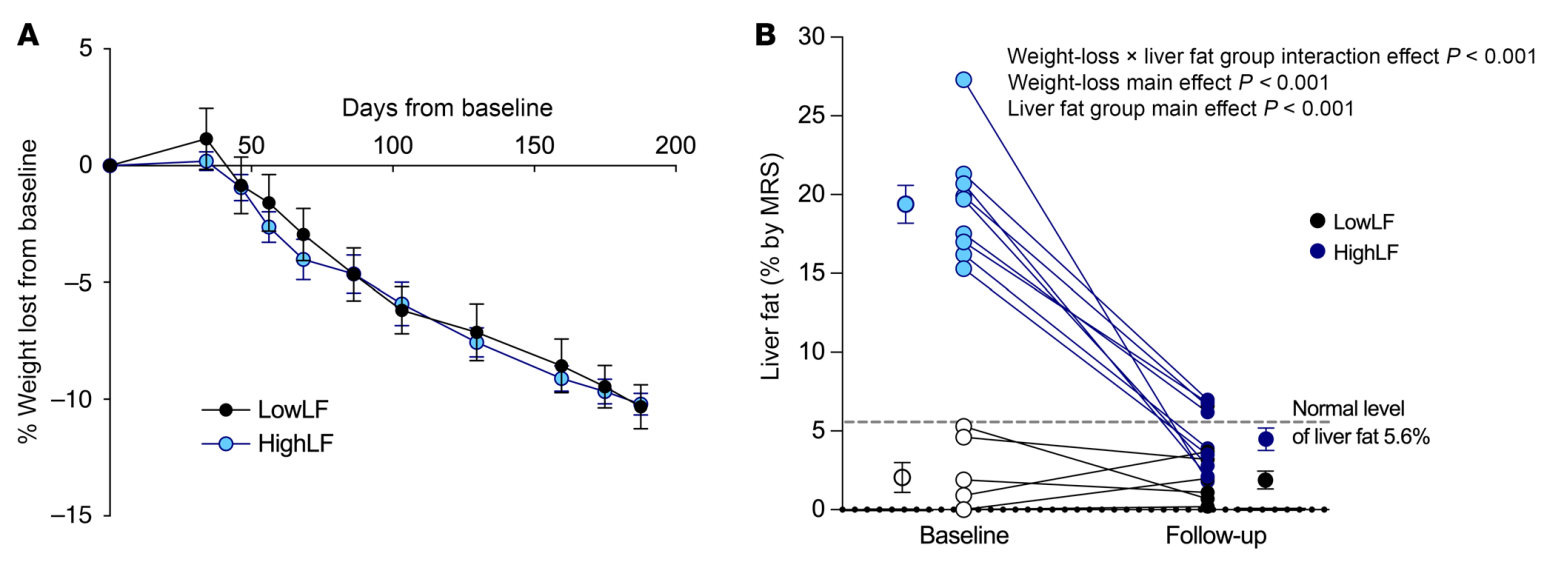
Weight loss and its impact on liver fat. (**A**) Weight loss trajectories of subjects with low liver fat (LowLF; *n* = 6) and high liver fat (HighLF; *n* = 9) over the 6-month dietary intervention; data are mean ± SEM. (**B**) Changes in liver fat measured by MRS. Change in liver fat between the groups due to weight loss was assessed by 2-way repeated-measures ANOVA.

Table 1 shows the dietary composition of the subjects at baseline and at the end of the 6-month intervention. Reductions in overall energy intake and improvement in dietary quality brought about reductions in the intake of all macronutrients (fat, protein, carbohydrate, and sugars), while fiber intake was maintained. Overall, the composition of the subjects’ diets was characterized by a reduction in percentage energy from fat (37% ± 2% at baseline to 31% ± 2% at follow-up), total sugars (21% ± 2% to 17% ± 1%), and added sugars (14% ± 2% to 6% ± 1%), while the energy from protein increased (17% ± 1% to 23% ± 1%). The effects of the stepwise and systematic reduction in food intake can be observed through the corresponding progressive loss of weight in Figure 2A. Similar significant reductions in body weight of about 10% occurred in both LowLF and HighLF groups (Figure 2A and Table 1). The main effect of weight loss (*P* < 0.001) was not affected by a liver fat group interaction (*P* = 0.229). Weight loss resulted in a significant 75.6% ± 4.0% relative reduction in IHTG in the HighLF group (*P* < 0.001 for a significant interaction effect of weight loss × liver fat group; Figure 2B) and resulted in normalization of IHTG (<5.6%) in the majority of subjects. The lowering of IHTG in the HighLF group to levels similar to those in the LowLF group allowed us to determine whether the 2 groups had similar fluxes of liver fatty acids at follow-up. Liver enzymes fell significantly with weight loss in both groups (Table 1). Of the 9 HighLF subjects, 4 subjects underwent diagnostic (medically indicated) liver biopsies at baseline and also had repeat biopsies after weight loss. Results from these subjects are included in Supplemental Figure 2 to show the effect of weight loss on liver injury. For example, subject 4 with mild liver injury at baseline (Supplemental Figure 2A) experienced an 11% weight loss, which was associated with a doubling of insulin sensitivity and reductions in alanine transaminase (–83%), plasma TGs (–50%), and liver fat (27.3% to 1.8%, assessed by magnetic resonance spectroscopy [MRS]) along with histological evidence of resolution of steatosis and fibrosis.

### Improved fasting and post-meal whole-body metabolism.

During admission 2 (Figure 1B), subjects consumed a standardized dinner at 1800 hours on day 1, followed by fasting until noon (1200 hours) on day 2. This protocol was used for 2 reasons: it allowed us to test the ability of the subjects to adapt to an acute extended fast and allowed enough time for stable isotope labeling of VLDL-TG to come to a steady state. At 1200 on day 2, a lunch meal identical to the evening dinner (same composition and energy) was fed, and additional blood samples were taken until 1800 to assess metabolic responses to eating after an extended fast. Four phases of the 24-hour test duration will be described in terms of changes in metabolism observed through transitioning between fasting and fed states across the 4 study time periods (post-dinner, night, extended fast, and lunch).

**Figure 3:**
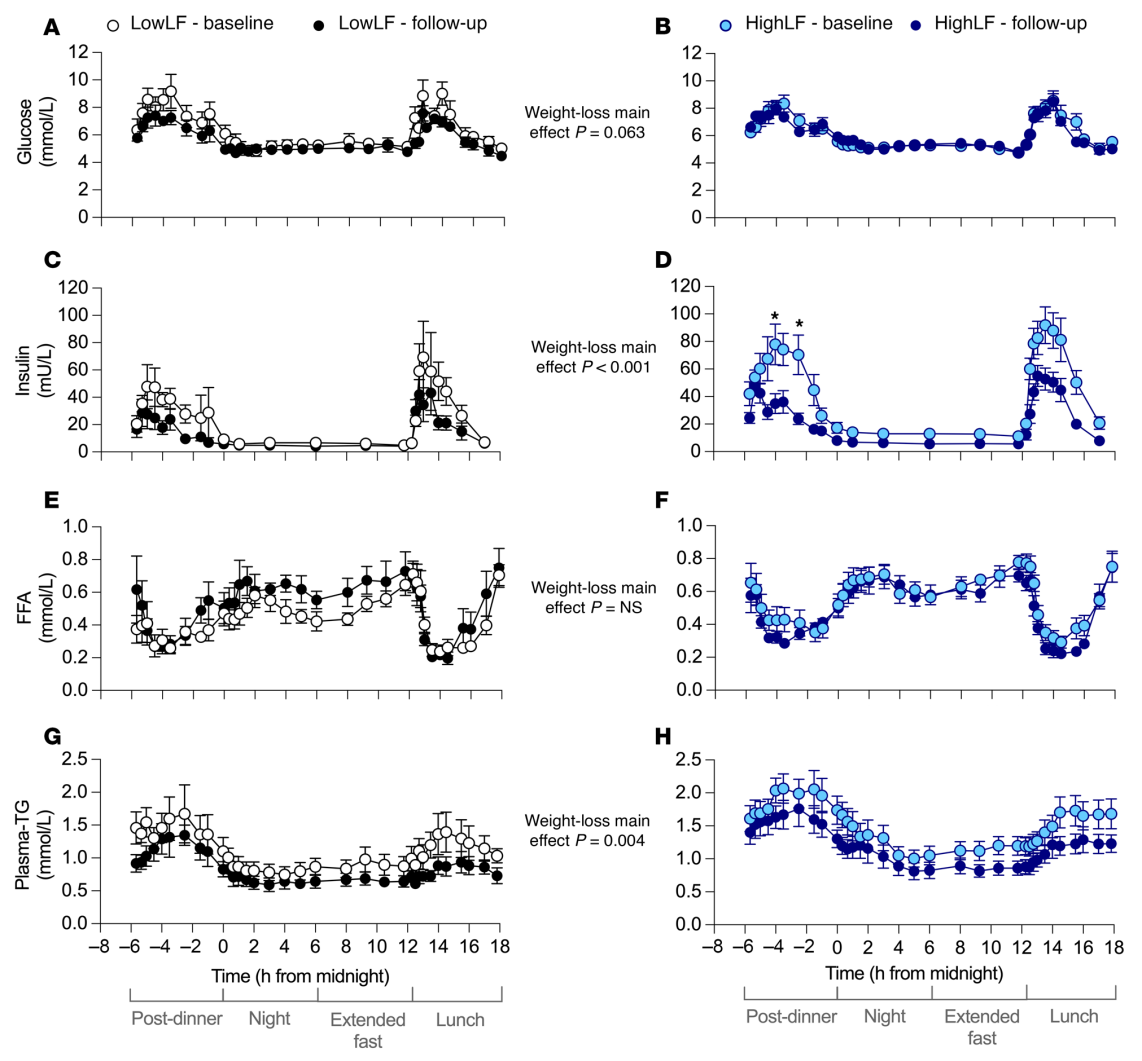
Plasma glucose, insulin, FFA, and TG levels after dinner, at night, and after an extended fast and lunch, before and after weight loss. Data are mean ± SEM for LowLF (*n* = 6) and HighLF (*n* = 9) for plasma levels of glucose (**A** and **B**), insulin (**C** and **D**), FFAs (**E** and **F**), and TGs (**G** and **H**). *P* values denoted on the graphs represent main effect of weight loss analyzed by 3-way RM-ANOVA for changes in metabolites by (i) time over the 24-hour testing period, (ii) weight loss, and (iii) liver fat group, with subgroup comparisons conducted for within-group changes after weight loss at each time point. *Time points significantly different after weight loss within a group.

Circadian responses of plasma glucose, insulin, FFAs, and TGs are shown in Figure 3, while fasting levels are summarized in Supplemental Figure 3. Circadian responses were analyzed by 3-way repeated-measures ANOVA (RM-ANOVA) for changes in metabolite concentrations in order to assess (a) the pattern and fluctuation of metabolites between fasting and fed states, (b) the effects of weight loss, and (c) any differences in observed effects by liver fat group. Glucose (Figure 3, A and B) increased following the dinner meal and progressively fell, plateaued during the night and the extended fasting period, and then rose following the lunch meal before progressively falling again (*P* < 0.001 for main effect of time course). Fasting glucose concentrations were not significantly different following weight loss (Supplemental Figure 3A). There was a tendency for weight loss to reduce plasma glucose concentrations over the 24-hour period (*P* = 0.063 for effect of weight loss).

Insulin concentrations (Figure 3, C and D) rose following the dinner meal and progressively fell, plateauing overnight and for the extended fasting period, and then rose again following the lunch meal before falling back to fasting levels. Weight loss significantly reduced plasma insulin concentrations in both groups, particularly in the fed states following the meals (post-dinner and lunch periods) (*P* < 0.001 for time course × weight loss interaction; Figure 3, C and D). After weight loss, lower insulin peak heights following the meals and a faster return to baseline demonstrate that the effect of weight loss was to chiefly impact the postprandial state. This finding is consistent with the improved sensitivity assessed by the i.v. glucose tolerance test (admission 1) observed in both groups (Table 2). Fasting insulin concentrations were significantly lower after weight loss in both groups (*P* = 0.002; Supplemental Figure 3B). A greater reduction in insulin was observed in the HighLF group (*P* = 0.075 for weight loss × liver fat group interaction effect for fasting insulin, and *P* < 0.001 for change from baseline to follow-up in the HighLF). The reduction in HighLF FFAs, along with a 50% reduction in fasting insulin, resulted in a significantly greater reduction in AdipoIR (Table 2). Both groups exhibited improvement in HOMA-IR (reflecting whole-body insulin resistance) and the disposition index (reflecting insulin secretion relative to action), and while the insulin sensitivity index rose in both groups, it tended to rise more in the LowLF group (Table 2).

Extended elevations in nighttime FFAs have been implicated in insulin resistance (30–32). Plasma FFA concentrations (Figure 3, E and F) fell after the meal as a result of insulin suppression of adipose lipolysis and then progressively rose, plateauing overnight and through the extended fast, before falling again in response to lunch. These circadian patterns were observed with no significant effect of weight loss (Figure 3, E and F), though overnight FFA concentrations tended to increase in the LowLF group after weight loss compared with HighLF (*P* = 0.071 for weight loss × liver fat group interaction). Fasting FFA concentrations tended to fall in the HighLF group (*P* = 0.037; Supplemental Figure 3C) but did not change in the LowLF group.

Finally, plasma-TG concentrations rise postprandially as a result of influx of chylomicrons carrying TGs from meal fat before progressively falling as they are cleared by lipolysis at peripheral tissues. Remnants are taken up by the liver. Here, overall plasma-TG levels were significantly lower in both groups after weight loss (Figure 3, G and H), resulting in reduced fasting plasma TG concentrations at follow-up (*P* = 0.006 for main effect of weight loss; Supplemental Figure 3D) and particularly in the HighLF group (*P* = 0.004). As shown in Figure 3, G and H, pre-dinner concentrations of plasma-TG were reduced with weight loss (as seen at the –6 hour time point on the graph), and plasma lipemia after both dinner and lunch was significantly lower after weight loss, though the LowLF group demonstrated a faster return to fasting levels (*P* = 0.056 for time course × liver fat group interaction).

**Figure 4:**
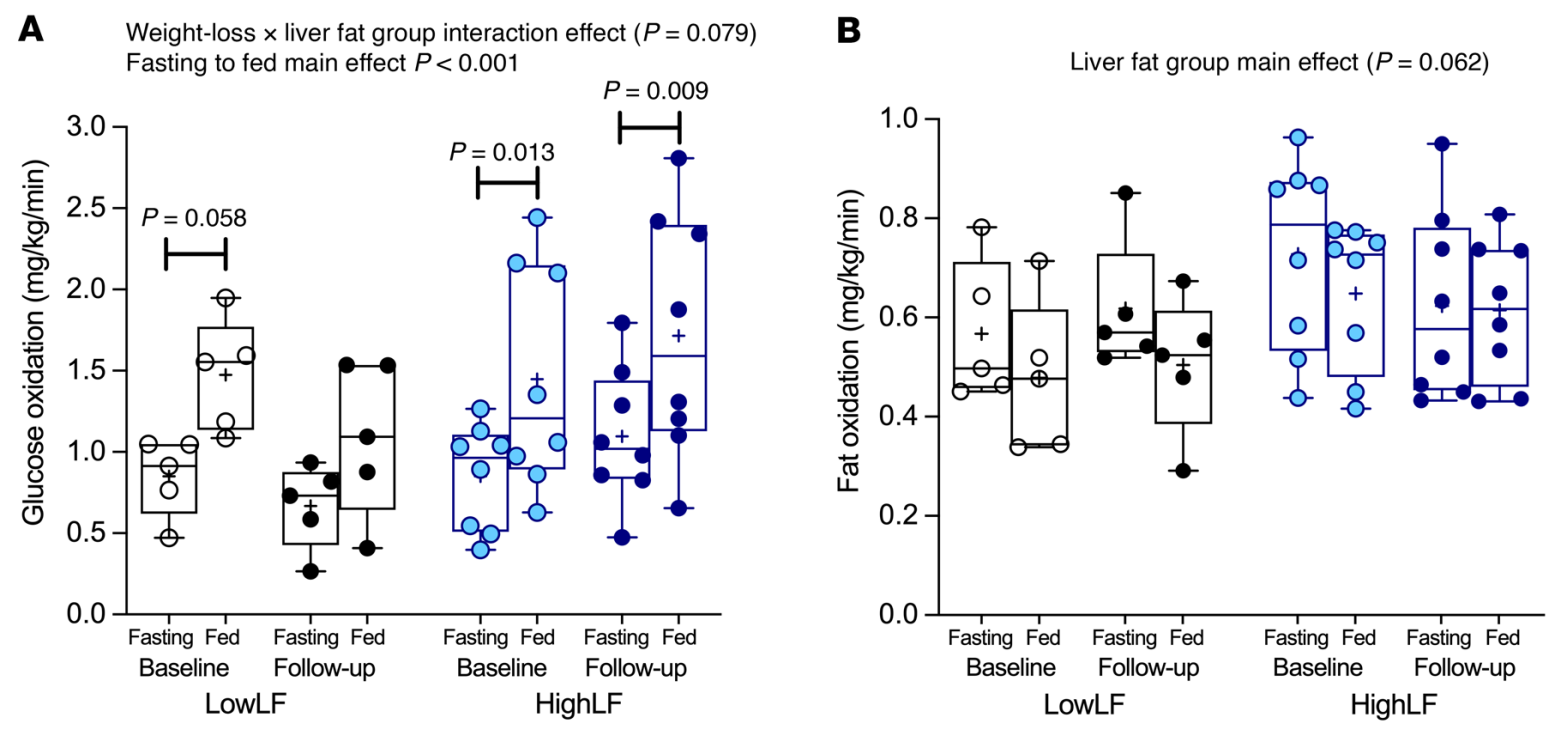
Glucose and fat oxidation in the fasting and fed states before and after weight loss. Respiratory gas analysis was conducted in the fasting and fed states to assess changes in glucose (**A**) and fat (**B**) oxidation. Three-way RM-ANOVA was used to assess the effects of (i) transitioning from fasting to fed states, (ii) weight loss, and (iii) liver fat group on changes in glucose and fat oxidation. *P* values above the graphs reflect main effects of fasting to fed state, weight loss, or liver fat group, and interaction effects of these. *P* values on the graphs represent change from fasting to fed state assessed through subgroup comparisons in the 3-way RM-ANOVA. Data are from LowLF and HighLF subjects showing the mean (indicated by “+”; may be behind a data point); boxes represent the 25th and 75th percentiles; middle line in the boxes represents the median; and whiskers represent the minimum and maximum values. One LowLF subject at baseline and one HighLF subject at follow-up had missing data (due to equipment malfunction), and therefore LowLF *n* = 5 and HighLF *n* = 8 for both fat and glucose oxidation measurements.

Respiratory gas analysis was used to assess whether fasting to fed state changes in glucose and fat oxidation were different following weight loss (Figure 4). Compared with the HighLF group, glucose oxidation was lower in the LowLF group, in both the fasting and fed states, after weight loss (*P* = 0.079 for weight loss by liver fat group interaction; Figure 4A). In LowLF subjects at baseline, the lunch consumption increased glucose oxidation 73% (from 0.85 to 1.47 mg/kg/min, *P* = 0.058), and after weight loss, the meal-induced change was similar (63%). In the HighLF group, meal-induced glucose oxidation rose 70% at baseline (*P* = 0.013), and after weight loss this rise was 56%; however, stimulation was more significant as a result of less variability between subjects (*P* = 0.009). Fed-state fat oxidation was higher in the HighLF group than in the LowLF at baseline and tended to remain higher after weight loss (main effect of liver fat group *P* = 0.062).

### Adipose fatty acid release.

**Figure 5:**
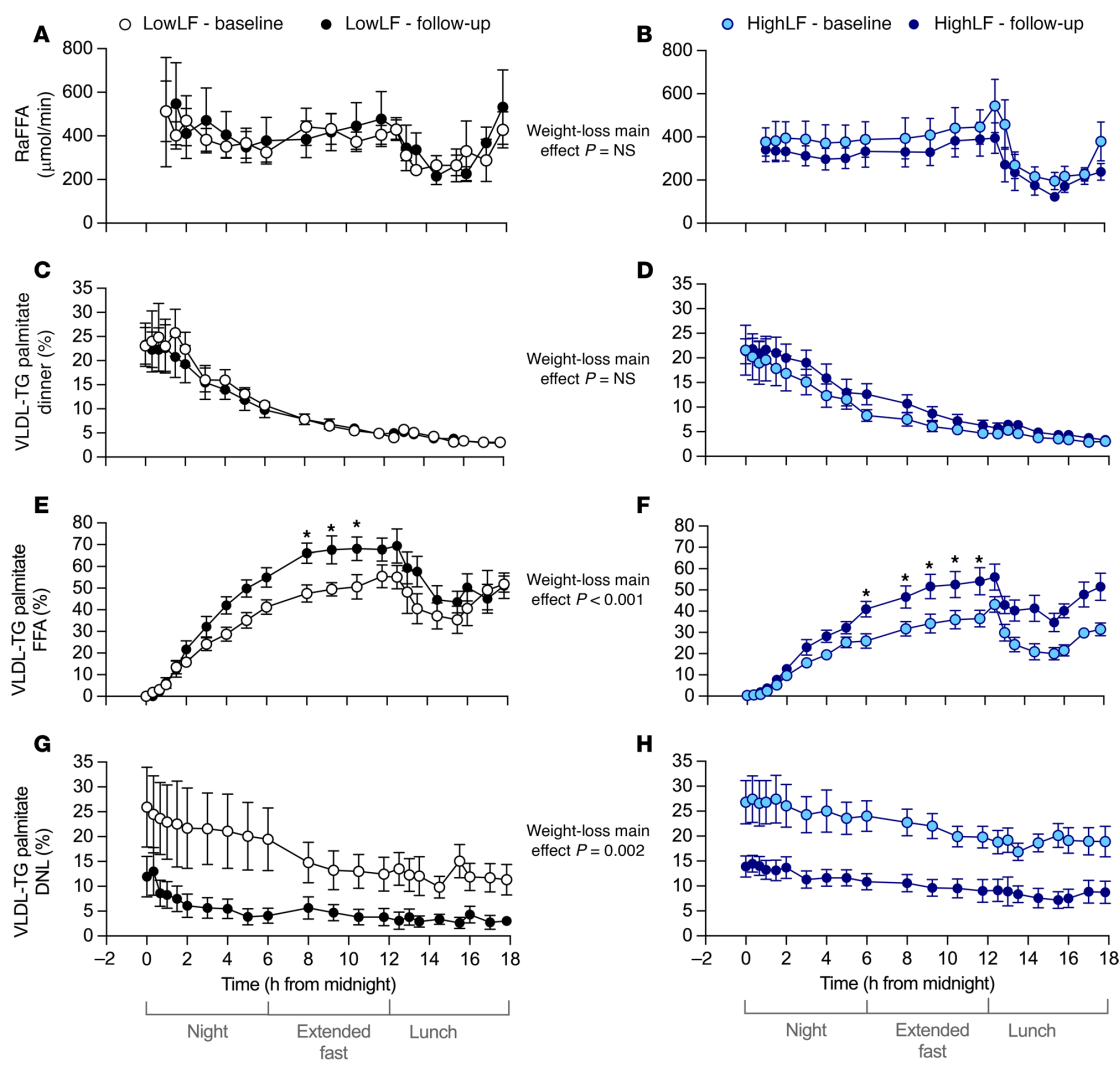
Plasma turnover of adipose-derived FFAs and labeled palmitate appearing in VLDL-TG based on the palmitate source before and after weight loss in LowLF and HighLF subjects. Isotopic labeling of the FFA pool was initiated at midnight. The rate of appearance of plasma FFAs (RaFFA; **A** and **B**) and proportions of VLDL-TG palmitate derived from the 3 sources (dinner meal, **C** and **D**; plasma FFA pool, **E** and **F**; and DNL, **G** and **H**) are presented from midnight onward. For the reader’s interest, the changes in proportional contributions of fatty acid sources after lunch are presented, but because this is not a system in steady state, statistical analysis of changes in sources (as well as RaFFA, for consistency) after lunch (1200 hours) was not performed. Therefore, *P* values on the graph represent main effect of weight loss analyzed by 3-way RM-ANOVA for changes in RaFFA and sources of VLDL-TG palmitate by (i) time over the 12-hour testing period (midnight to 1200 only), (ii) weight loss, and (iii) liver fat group, with subgroup comparisons for within-group changes after weight loss at each time point. Data are mean ± SEM for LowLF (*n* = 6) and HighLF (*n* = 9). *Time points significantly different within a group after weight loss.

Stable isotopes were administered for quantitation of plasma FFA turnover and metabolism, and palmitate was used as the representative fatty acid to assess contributions from adipose-, dietary-, and lipogenesis-derived fatty acid sources (Figure 1). The Methods section details the multiple components used to calculate the rate of appearance of plasma FFAs from adipose tissue (RaFFA). Three sources of plasma FFAs were measured: (a) FFAs released from intra-adipose lipolysis, (b) FFAs liberated from LPL-mediated lipolysis of chylomicrons, and (c) LPL-mediated lipolysis of VLDL in the plasma. The RaFFA data presented in Figure 5, A and B, represent the adipose release only. No effects of weight loss on RaFFA during the night or extended fasting periods (0000–1200 hours) were observed (*P* = 0.562 for main effect of weight loss, and no effect of liver fat group).

### Fatty acid sources used for TG synthesis.

We have shown previously that the fatty acid sources found in VLDL-TG (diet, FFAs, and DNL) reflect the contributions to TG esterification within the liver (12, 14, 23, 24, 33). Here, the sources were calculated based on their proportional contribution to fasting VLDL-TG palmitate, and these graphs reflect the origin of the fatty acid (Figure 5, C–H). In addition to the nocturnal patterns of labeling presented in Figure 5, the changes in fatty acid sources after lunch (1200 hours) are also included for the reader’s interest; however, because this is not a system in steady state owing to influx of chylomicrons following the meal, statistical analysis of changes in sources was not performed after lunch.

In Figure 5, C and D, no difference was observed between groups in the appearance of dietary fat in VLDL-TG and no effect of weight loss. Figure 5, E and F, demonstrates that weight loss was associated with a greater proportion of fatty acids from the FFA pool appearing in VLDL-TG palmitate in both groups all night long (*P* < 0.001). In addition, weight loss appeared to cause the LowLF group to reach a plateau of the fractional contribution of FFAs to VLDL-TG earlier in the night. Lastly, at baseline, the proportion of VLDL-TG palmitate arising from DNL was higher in both groups during the night (starting at ~25%–30% at midnight; Figure 5, G and H), and then it progressively fell overnight and through the extended fast. Weight loss significantly reduced DNL in both groups in the postprandial state (midnight time point) and throughout the night (*P* = 0.002 for main effect of weight loss; Figure 5, G and H). After weight loss, the LowLF group exhibited a very strong suppression of lipogenesis such that it reached levels below 5% by 0200 hours.

**Figure 6:**
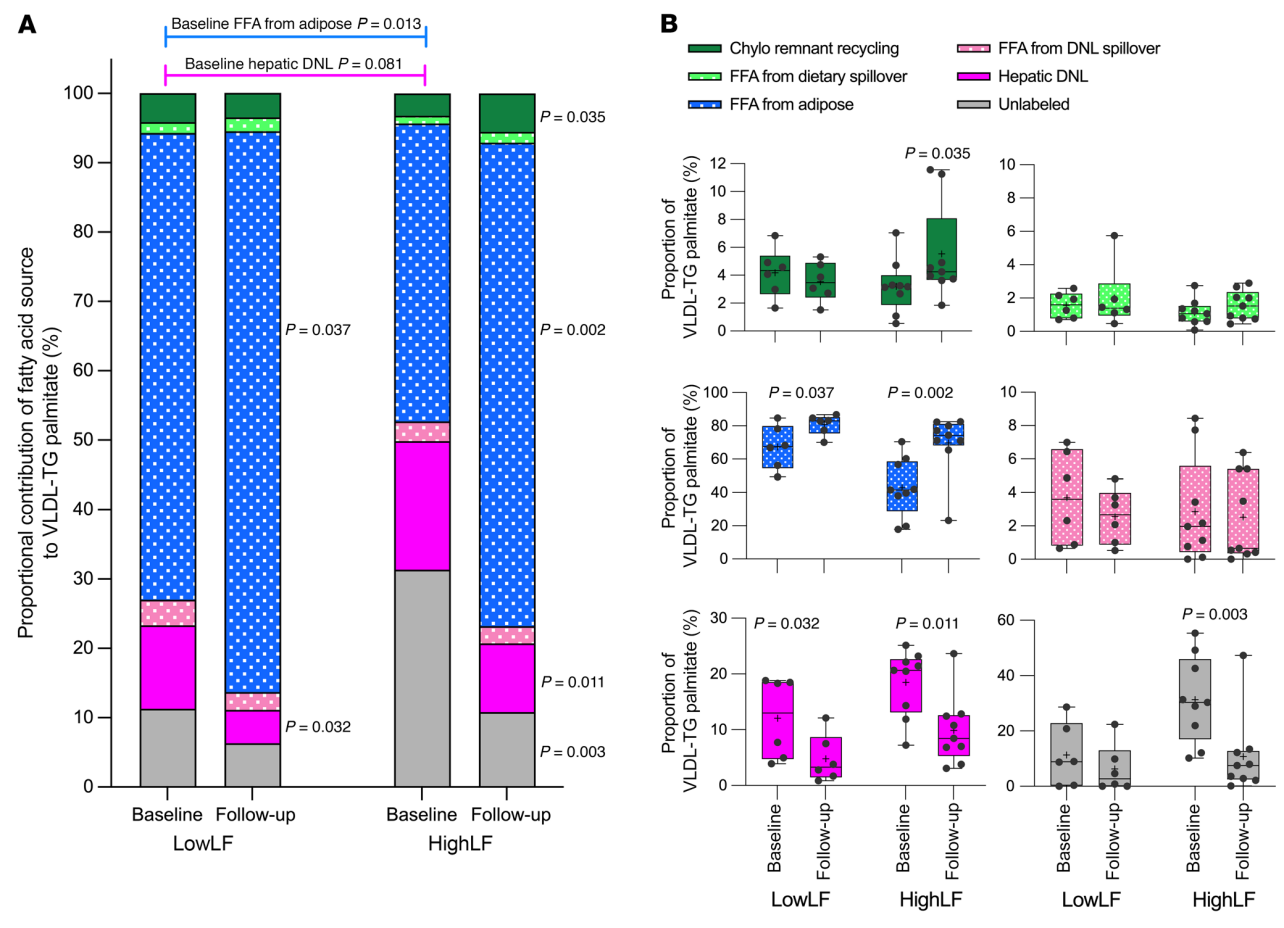
Six routes of palmitate entry into hepatic VLDL-TG assembly. (**A**) Data are means, with variances removed for clarity, for LowLF (*n*= 6) and HighLF (*n* = 9). (**B**) Individual data points are shown with the mean (indicated by “+”; may be behind a data point); boxes represent the 25th and 75th percentiles; middle line in the boxes represents the median; and whiskers represent the minimum and maximum values. At the end of the fasting period (before the lunch meal), sources of palmitate were quantitated as a proportion found in VLDL-TG. Proportional contributions reflect the contribution of fatty acid sources being used within the liver at the site(s) of intracellular-TG synthesis as reflected in VLDL. A portion of VLDL-TG is synthesized from stored intrahepatic lipid that does not become labeled during the course of isotope administration (gray bars). Changes due to weight loss were assessed between the groups using 2-way RM-ANOVA. There were main effects of weight loss (*P* < 0.001) and liver fat group (*P* = 0.025) for proportion of VLDL-TG palmitate derived from FFAs as well as for DNL (weight loss *P* = 0.001 and liver fat group *P* = 0.041). *P* values above the bar graphs in **A** show differences between the 2 groups at baseline (assessed by unpaired 2-tailed *t* test). *P* values next to the bars in **A** represent baseline versus follow-up comparisons within a single group (assessed by paired 2-tailed *t* test); these within-group comparisons are reiterated above the bars in **B**.

The temporal patterns of labeling are shown in Figure 5, while Figure 6 provides more detail on how those labels reached the liver and were used for VLDL-TG synthesis. For instance, dietary label can enter the liver via receptor-mediated uptake of chylomicron remnants, or, when chylomicrons undergo LPL-mediated lipolysis in the plasma, the released fatty acids can end up on albumin (e.g., contribute to the FFA pool in a process referred to as “spillover”). Spillover fatty acids contribute to FFA flux. In Figure 6, these 2 routes of dietary fat usage have been delineated (both in green). In the HighLF subjects only, weight loss increased the appearance of remnant uptake into the liver and recycling of this lipid through VLDL-TG synthesis, accounting for 3.2% ± 0.6% at baseline to 5.5% ± 1.2% at follow-up (*P* = 0.035). By contrast, FFA from spillover, which provided between 1.1% and 5.8% of VLDL palmitate, was not affected by weight loss in either group. With regard to the proportions of FFAs derived from adipose tissue found in VLDL-TG, these contributions were significantly increased after weight loss — from 67.3% ± 5.4% to 80.9% ± 2.5% in the LowLF group (*P* = 0.037), and from 42.9% ± 5.8% to 69.7% ± 6.1% (*P* = 0.002) in the HighLF group (Figure 6). The increase in proportional use of plasma FFAs for VLDL-TG indicated a greater relative hepatic esterification of plasma FFAs that were used for VLDL-TG export. Indeed, the rate of FFAs incorporated into VLDL-TG, calculated as a proportion of the available FFAs in plasma (RaFFA) that are taken up by the liver, showed a trend toward increasing in both groups after weight loss (LowLF 13.6% ± 2.4% to 19.0% ± 4.0% and HighLF 14.1% ± 2.3% to 20.0% ± 3.6%, *P* = 0.053 for main effect of weight loss).

Since lipogenesis was labeled over 10 days in this experiment, LPL-mediated lipolysis of labeled VLDL-TG DNL could spill over DNL fatty acids into the plasma FFA pool. We did detect small amounts of DNL-derived fatty acids in the plasma FFA (herein referred to as FFA-DNL) and thus accounted for their appearance in VLDL-TG (Figure 6). In the LowLF group, at baseline and follow-up, similar levels of VLDL-TG palmitate were derived from FFA-DNL (3.7% ± 1.1% vs. 2.6% ± 0.7%, respectively, *P* = 0.091). In the HighLF group, these percentages were also not different between baseline and follow-up (2.9% ± 1.1% vs. 2.5% ± 0.9%, respectively, *P* = 0.750). Within VLDL-TG, the majority of de novo fatty acids that were directly used for intrahepatic-TG synthesis were derived from hepatic DNL. Weight loss significantly reduced hepatic DNL in both the LowLF group (baseline 12.0% ± 3.0% vs. follow-up 4.8% ± 1.7%, *P* = 0.032) and the HighLF group (baseline 18.5% ± 2.0% vs. follow-up 9.9% ± 2.1%, *P* = 0.011).

Lastly, a portion of VLDL-TG is synthesized from lipid that does not become labeled during the duration of isotope administration. For the HighLF group, the unlabeled portion was significantly reduced (*P* = 0.003; Figure 6), which is consistent with the concept that the unlabeled portion emanates from IHTG stores, which were also significantly reduced in this group (Figure 2). Comparing the 2 liver fat groups directly, at the beginning of the study, the LowLF group had a significantly greater proportion of FFAs from adipose used for VLDL-TG synthesis compared with the HighLF group (67.3% ± 5.4% vs. 42.9% ± 5.8%, *P* = 0.013; Figure 6) and tended to have lower DNL (12.0% ± 3.0% vs. 18.5% ± 2.0%, *P* = 0.081). Weight loss produced similar changes in the proportional contributions to VLDL-TG palmitate in both groups, characterized by an increase in the use of adipose-derived FFAs and a reduction in DNL. Overall, the HighLF group exhibited a greater magnitude of changes with weight loss, which resulted in a normalization of all hepatic sources used to make VLDL-TG such that at follow-up there was no difference between the groups.

### Absolute contributions of lipids in VLDL-TG palmitate reflect reduced hepatic DNL and increased FFA re-esterification.

When the proportional values of the 6 sources are multiplied by the total VLDL-TG palmitate concentration (12, 24), the absolute concentration of fasting sources in VLDL-TG palmitate can be used to assess the causes of hyperlipidemia present in MASLD (Supplemental Figure 4). Similarly to the fractional sources, the absolute sources of VLDL-TG palmitate were normalized in the HighLF subjects to make them not different from the amounts in the LowLF group after weight loss. The absolute change in IHTG was associated with a significant reduction in hypertriacylglycerolemia (*r* = 0.531, *P* = 0.042; Supplemental Figure 5A), a reduction in the amount of unlabeled fatty acids (i.e., identification of greater amounts of palmitate in VLDL-TG) (*r* = 0.762, *P* = 0.010; Supplemental Figure 5B), and lower TG-palmitate from DNL (*r* = 0.650, *P* = 0.009; Supplemental Figure 5C). None of the other fatty acid sources in VLDL-TG were associated with IHTG reduction.

### Peripheral TG metabolism.

**Figure 7:**
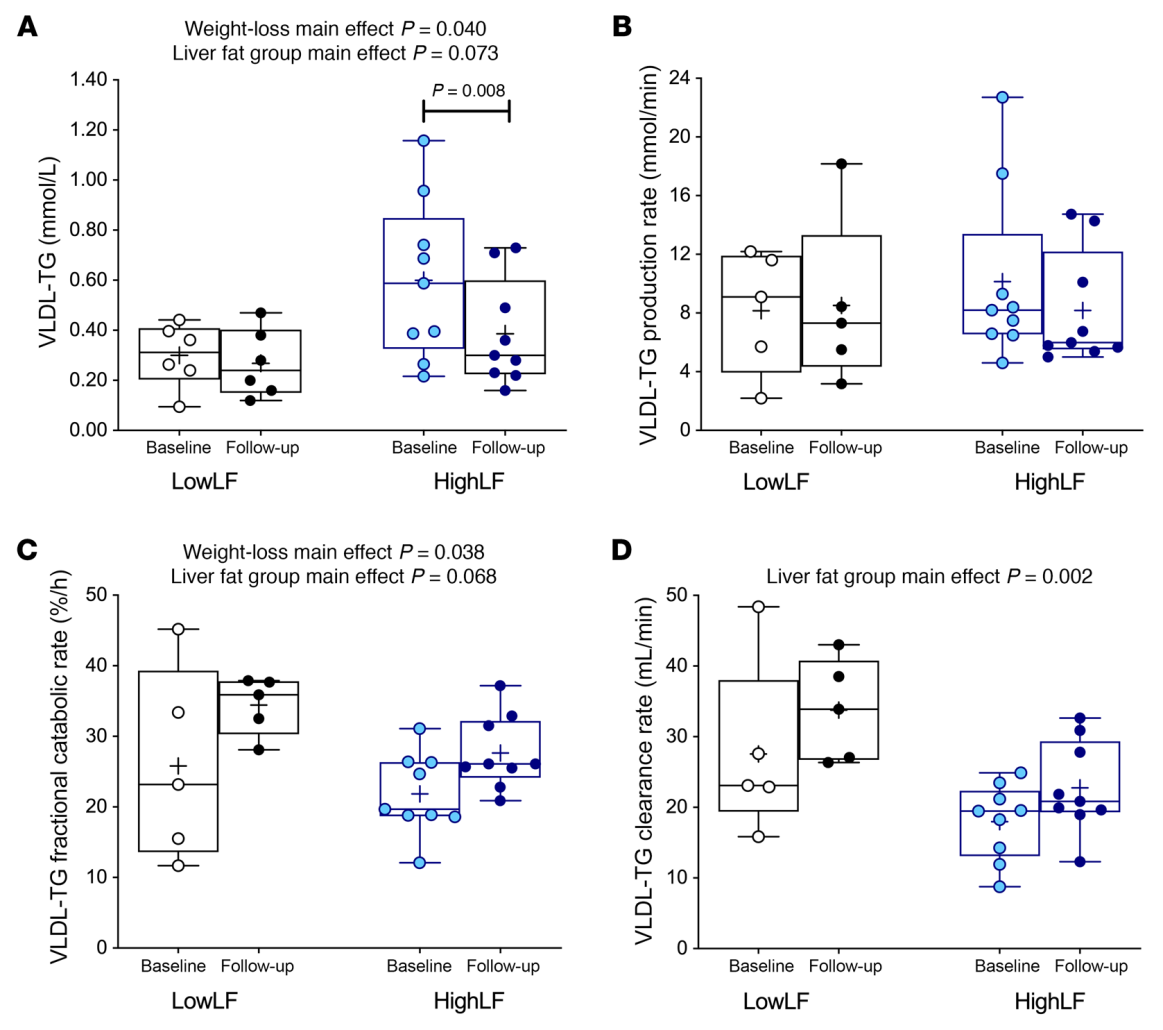
VLDL-TG concentrations and turnover rates before and after weight loss. Data are from LowLF subjects (*n* = 6 for **A** and *n* = 5 for **B**–**D** because of missing data for 1 subject that precluded kinetic analysis) and HighLF subjects (*n* = 9) and show impacts of weight loss. (**A**) Reduction of fasting VLDL-TG concentrations. (**B**) No changes in VLDL-TG production rates. (**C**) Increase in fractional catabolic rates. (**D**) Modest effect on particle-TG clearance rate. Data are presented as mean (indicated by “+” on the graph; may be behind a data point); boxes represent the 25th and 75th percentiles; middle line in the boxes represents the median; and whiskers represent the minimum and maximum values. *P* values above the graphs indicate main effects of weight loss and liver fat group determined by 2-way RM-ANOVA; no interaction effects were observed. *P* values on the graphs represent subgroup comparisons from the 2-way RM-ANOVA in the change from baseline to follow-up within LowLF and HighLF groups.

**Figure 8:**
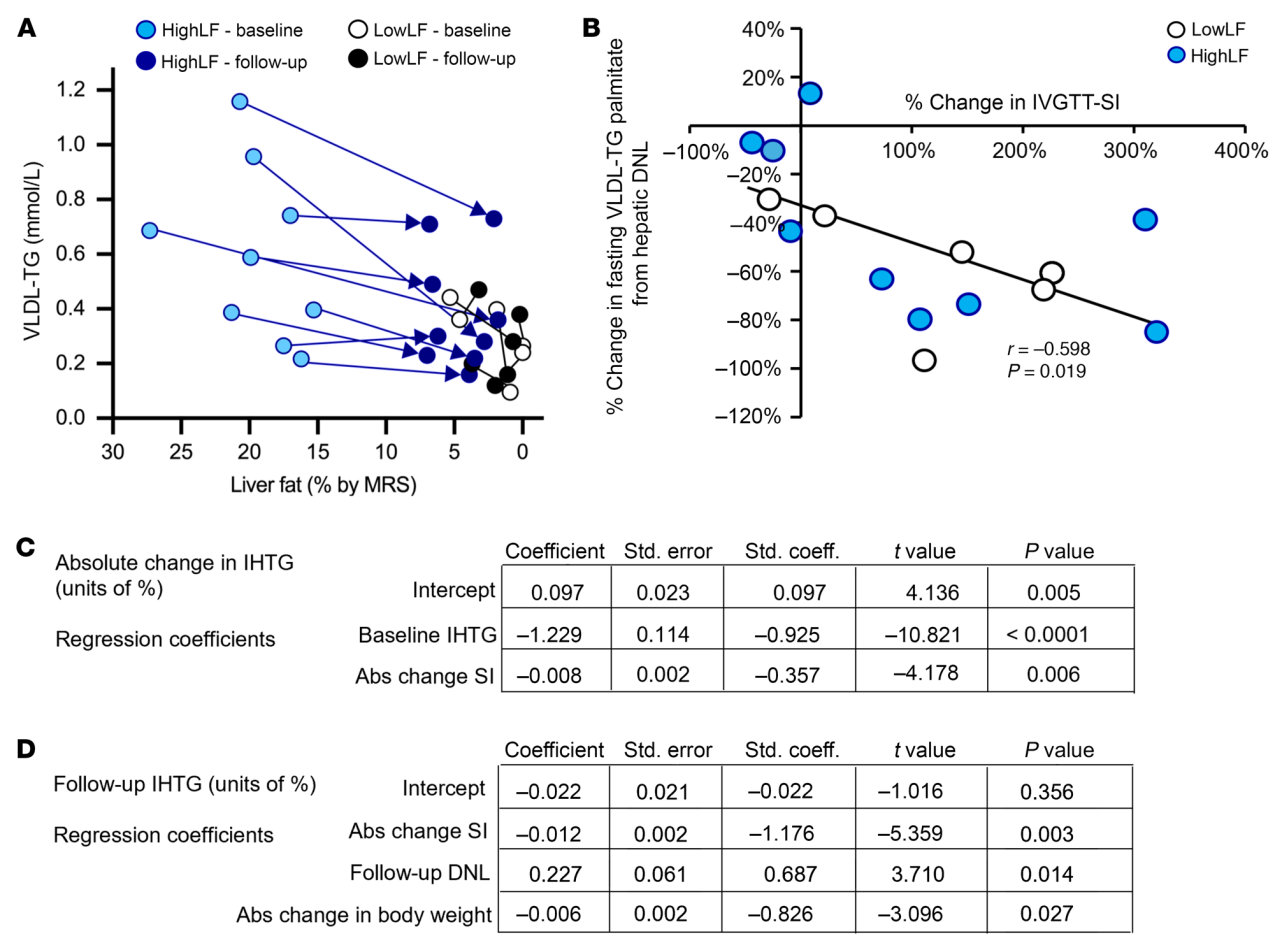
Relationships between changes in liver fat and reductions in VLDL-TG, insulin sensitivity, and lipogenesis. (**A**) Arrows depict the magnitude of absolute changes in liver fat and plasma VLDL-TG concentrations for HighLF (*n* = 9) and LowLF (*n* = 6) subjects. (**B**) For all subjects combined, the greater the improvements in insulin sensitivity (assessed by the insulin sensitivity index via the i.v. glucose tolerance test), the greater the reductions in fasting VLDL-TG palmitate %DNL (assessed by Pearson’s correlation). For the HighLF subjects, stepwise regression coefficients are presented for predictors of the absolute (Abs) change in IHTG with weight loss (**C**), and for predictors of the final level of IHTG at follow-up (**D**). For example, in **C**, large reductions in IHTG (follow-up minus baseline) were predicted by greater baseline IHTG levels and greater increases in insulin sensitivity.

Finally, weight loss reduced fasting VLDL-TG concentrations (*P* = 0.040; Figure 7A), particularly in the HighLF group (*P* = 0.008). This reduction in VLDL-TG concentration was not due to a reduction in VLDL-TG production rate (Figure 7B), but primarily to increased VLDL-TG fractional catabolic rate (*P* = 0.038; Figure 7C), with a modest increase in clearance rate (*P* = 0.126 for weight loss; Figure 7D). Across all subjects (Figure 8A), while liver fat was uniformly reduced (as much as 12-fold in some subjects), VLDL-TG concentrations were reduced by a lesser amount (2-fold). These data suggest that the magnitude of liver fat content is more tied to hepatic nutrient overload — a factor that can be acutely modified — while only a proportion of baseline hypertriacylglycerolemia can be mitigated by reducing energy intake. n univariate analysis, the improvements in insulin sensitivity were significantly associated with reductions in the fraction of VLDL-TG palmitate that was derived from DNL (*r* = –0.598, *P* = 0.019; Figure 8B). In multiple regression analysis, data from the HighLF group showed that IHTG change was predicted by a higher baseline IHTG (*P* < 0.0001) and greater absolute improvements in the insulin sensitivity index (*P* = 0.006) (adjusted *R*^2^ = 0.942, *F*_2,6_ = 65.5, *P* < 0.0001; Figure 8C). After weight loss, higher residual IHTG was predicted by no or low improvements in the insulin sensitivity index (*P* = 0.003), a lower loss of body weight (*P* = 0.027), and a higher level of DNL at follow-up (*P* = 0.014), with post–weight loss DNL being the strongest predictor of remaining IHTG (adjusted *R*^2^ = 0.849, *F*_3,5_ = 16.0, *P* = 0.005; Figure 8D).

## Discussion

This study used a comprehensive testing protocol to investigate the impact of weight loss on multiple aspects of fatty acid and triglyceride metabolism in subjects with elevated liver fat (HighLF) and similarly insulin-resistant subjects with low liver fat (LowLF). We assessed the sources of IHTG, plasma FFA flux, whole-body substrate oxidation, and VLDL-TG kinetics, during fasted and fed states and throughout the night. In well-fasted, lean subjects, fatty acids derived from adipose tissue stores are the primary source of fatty acids used by the liver for VLDL-TG synthesis, while DNL typically represents about 5%–10% of these sources, and dietary contribution in the fasting state is usually minor (23, 24). In the present study, for those with HighLF, adipose FFAs contributed about 43%, and diet from all routes contributed about 4% (1% from chylomicron fatty acid spillover into the FFA pool, and 3% calculated to come liver from chylomicron remnant uptake). DNL contributed about 21% to VLDL-TG palmitate when the sum of hepatic DNL directly used for VLDL-TG synthesis (19%) and DNL fatty acids entering the liver through the FFA pool (2%) were accounted for (Figure 6). After weight loss, the proportions of all 6 sources were the same between the groups, characterized by the predominant source of fatty acids for VLDL-TG being derived from adipose lipolysis (74% for all subjects combined) with lower amounts derived from diet (7%) and DNL (10%).

### Alterations in the sources of palmitate in VLDL-TG and effect of weight loss.

Given that adipose FFAs are the major source of fatty acids for hepatic lipids, it has been suggested that increased availability of plasma FFA flux is a main driver of liver fat accumulation in MASLD (9). Further, previous data have shown that the nighttime rate of appearance of plasma FFAs (RaFFA) is elevated in states of insulin resistance (30–32). However, at baseline, no group differences were observed in fasting plasma FFA concentrations or FFA delivery (RaFFA; Figure 5), and weight loss did not result in changes in RaFFA, though fasting plasma FFA concentrations were reduced in the HighLF group (Supplemental Figure 3C). By contrast, the proportional contribution of FFAs to VLDL-TG was greater following weight loss, and the rates at which plasma FFAs were recycled into VLDL-TG that was secreted were also greater. These data suggest that a reduction in liver re-esterification and storage of FFAs was one mechanism contributing to the reduction in IHTG. Given that FFA oxidation is reduced by malonyl-CoA (a by-product when fatty acid synthesis is on), it is highly possible that lower malonyl-CoA increased both hepatic FFA oxidation and also routing of FFAs to TG secretion.

The DNL pathway is sensitive to excess substrate availability (fructose and glucose carbons) coupled with insulin resistance and can substantially contribute to hepatic lipid accumulation in both quantitative and qualitative ways (11, 34–36). We have recently shown that the level of DNL is higher in patients with progressively more advanced liver disease (14). Quantitatively, DNL contributes newly made fatty acids (11), and qualitatively, activation of the DNL pathway also upregulates genes involved in esterification and storage of fatty acids from any source (37). DNL is activated in the fed state and becomes suppressed during fasting, which can be observed here in the change in proportion of VLDL-TG palmitate from DNL in the LowLF subjects at baseline from midnight (time 0) to 0500 hours (Figure 5G) — a trajectory that was much slower in the HighLF subjects (Figure 5H). The importance of acute energy flux to the rate of DNL is exemplified by the fact that such marked weight loss–induced changes in DNL patterns can be observed, and yet these subjects still had obesity/overweight at the end of the study.

The intervention reduced overall total energy content of the diet, with specific focus on total and added sugars. Weight loss resulted in notable improvements in insulin sensitivity and glucose metabolism, observed in the HighLF subjects by a greater glucose oxidation in the fasting state and glucose disposal (Figure 7B). In the LowLF group, greater peripheral glucose clearance was coupled with lower fed-state glucose oxidation rates, supporting improved non-oxidative glucose disposal. In human metabolic studies, relationships between changes in physiological variables can be hard to detect, and findings this robust suggest that interventions that increase peripheral glucose oxidation should be vigorously advocated for MASLD patients (38–41).

The relative contributions of dietary fat to VLDL-TG palmitate in both the fed state (observed at midnight, 6 hours after the dinner meal) and the fasting state were similar between LowLF and HighLF groups at baseline, and surprisingly, in the HighLF group after weight loss, the liver appeared to take up chylomicron remnants and recycle the lipid to VLDL at a slightly higher rate (Figure 6). The proportion of fatty acids in VLDL-TG that remained unlabeled after isotope administration was reduced 66% after weight loss (*P* = 0.003; Figure 6). We interpret this result to be due to lower availability of hepatic-TG stores used for VLDL synthesis. Taken together, the data suggest that when DNL is elevated it exerts an additional lipid burden — it does not displace other fatty acid sources for inclusion into VLDL-TG but instead adds to them by increasing the re-esterification of all sources of lipid. This effect results in hepatic lipid excess and hypertriacylglycerolemia. If correct, this suggests that energy restriction and weight loss–induced reductions in nutrient overload in the liver are associated with (a) peripheral usage of glucose, which would serve to lower liver substrates available for DNL; (b) FFAs being routed away from hepatic storage and toward VLDL secretion; and (c) loss of previously stored hepatic TG, presumably depleted via increased hepatic oxidation.

The lack of increase in RaFFA may appear a paradox, because it might seem obvious that a principal way a person can lose body fat is if it comes out of the adipose tissue to be burned by the body. However, in the HighLF group, we estimated that the magnitude of the increase in daily adipose RaFFA needed to produce the observed 5.1 ± 0.8 kg reduction in adipose mass cover 6 months was 70 ± 11 μmol/min. This amount is small compared with the turnover of plasma FFAs (~300–400 μmol/min; Figure 5, A and B), and thus this change may be difficult to detect. Using the calculations of Magkos et al., our sample size should have been sufficient to detect such an increase in RaFFA (42). Another peripheral mechanism that would support loss of adipose depot sizes without a substantial change in RaFFA would be if the dietary treatment reduced adipose-TG synthesis. This mechanism was likely, given the lower insulin concentrations produced by weight loss, which would serve to reduce both intra-adipocyte lipogenic enzymes and lipid clearance to adipose tissue (43).

### VLDL kinetics in MASLD and after weight loss.

The increased cardiometabolic risk of patients with MASLD is characterized by an atherogenic phenotype (44, 45). Weight loss raised the HighLF subjects’ HDL-cholesterol by 14% (*P* = 0.045; Supplemental Table 1) and lowered plasma TG by 20% (*P* = 0.006) and VLDL-TG by 27% (Figure 7A), the latter occurring via a 35% increase in VLDL-TG fractional catabolic rates (Figure 7C). Other investigators have observed a reduction in VLDL-TG fractional clearance rate in individuals with obesity following weight loss via gastric bypass surgery (46) or in women with abdominal obesity on a low-energy diet (47); however, neither publication included measures of IHTG. In the present HighLF group, the magnitude of reductions in IHTG was large in comparison with reductions in VLDL-TG concentrations (Figure 8A). This highlights the fact that IHTG is strongly driven by acute energy restriction (48) whereas plasma lipid concentrations may be more genetically controlled (49, 50). Our data suggest that the ability of the periphery to adapt in a manner that reduces the stress on the liver protects against MASLD. In the fed state, better substrate disposal was evident after dinner in the observed lower levels of insulin needed to maintain glucose concentrations (39, 51, 52). In this scenario, rerouting of nutrients away from the liver, toward peripheral storage and oxidation, reduces hepatic toxicity.

### Limitations and future research needs.

The primary limitation of this study relates to the nature of investigations using measures of insulin sensitivity and isotopic labeling, which require intensive protocols that typically limit study sample sizes. Labeling of plasma FFAs and DNL is distinct from other work in the literature, which has had a focus on investigating either of these 2 sources separately (25, 46, 47, 53). Fasting VLDL-TG production rate was measured to assess synthesis during a steady state; the sources of VLDL-TG would likely be different if assessed in the fed state. Palmitate was used as the marker for all fatty acids because it is the primary product of DNL, the second most prominent fatty acid in the diet, and one of the main fatty acids in plasma FFAs and VLDL-TG. The contribution of DNL to other fatty acids is variable, and it would not contribute to essential fatty acids like linoleic acid. Therefore, DNL contribution to total fatty acids is challenging to estimate and is likely lower than what is observed for palmitate. For dietary intervention studies, many staff and resources were needed to support subjects and limit dropout rates over long durations (6 months of treatment), and this project had the additional subject burden of measuring nocturnal flux rates. It is slightly unusual that the weight loss trajectories of both groups remained nearly linear over the 6-month investigation, rather than falling precipitously at the beginning and slowing toward the end. The continued rate of weight loss was due to the dietitian emphasizing progressively greater reductions in food intake throughout the study. The final 3 weeks during post–weight loss testing, body weight was held steady to achieve a consistent metabolic state at the follow-up admission. Although longer than some intervention studies, the duration of 6 months for weight loss may not have been long enough to cause all subjects to reach IHTG levels below 5.5%.

The calculation of sources of VLDL-TG palmitate is based on a number of model assumptions described in Supplemental Methods. One characteristic of TG metabolism measured with isotopes is that it invariably results in some recycling of labels during data acquisition. This was evident by the presence of DNL fatty acids in the plasma FFA pool, which could have been derived from release of adipose fatty acids that were labeled by administration of heavy water or could have been derived from spillover of VLDL-DNL fatty acids. Given that 10 days is a relatively short duration of D_2_O administration to be able to detect labeling of adipose TG, we favor the latter explanation for the presence of DNL in FFAs. Absolute VLDL-TG concentrations from hepatic DNL and FFA-DNL were positively correlated (*r* = 0.852, *P* = 0.001 at baseline and *r* = 0.884, *P* = 0.002 at follow-up). A second unknown in the model is the source of unlabeled lipid in VLDL-TG. It could have resulted from unmeasured visceral dilution of the palmitate label as it entered the liver or from intrahepatic lipid droplets that slowly turned over, contributing unlabeled lipid to VLDL-TG synthesis. Our original study of lipid labeling in MASLD patients demonstrated a strong association between the magnitude of stored lipid and the unlabeled lipid in VLDL-TG (12). Given the present study’s weight loss intervention, both IHTG and visceral fat were likely reduced, and thus, the true source of the unlabeled pool cannot be definitively identified. Third, the study was not designed to understand the unique mechanisms that protected the LowLF group from fatty liver at baseline. Given that these subjects had similar levels of obesity and insulin resistance, understanding this protection would be important to accomplish. Measurement of lipogenesis in lean MASLD (54) should also be investigated to determine whether DNL is elevated in this population. It is our belief that both insulin resistance and genetic factors, rather than just excess body weight, are associated with MASLD. Lastly, although the HighLF subjects investigated here exhibited a considerable reduction in IHTG, their post–weight loss IHTG values were still greater than those of the LowLF group. Although genetic contributors to IHTG have been identified (55), no studies to date have tested the heritability of lipogenesis within families. Lifestyle factors (e.g., levels of dietary intake, exercise) can influence levels of IHTG, and the present study focused on energy restriction to lower hepatic nutrient overload. Additional work is needed to uncover how increases in energy expenditure through exercise may limit nutrient toxicity also (38, 56, 57) and improve the effect of energy restriction (58).

### Conclusions.

The present findings substantially advance the integrated understanding of fatty acid metabolism in the development of MASLD. When the contribution of all sources of fatty acids to IHTG was quantitated, it was excess lipogenesis that was the primary driver of liver fat. Insulin resistance, previously shown to be implicated in excess IHTG, was markedly improved with weight loss, and these improvements predicted loss of liver fat. Weight loss also substantially improved the observed baseline slow circadian transitions found after dinner and through the night as fasting was extended. In our previous baseline studies of subjects matched for insulin resistance, elevated DNL was found to be the singular variable segregating HighLF and LowLF subjects (11). Here, we show that when liver fat is substantially reduced by energy restriction, it is the reduction in lipogenesis that largely accounted for the liver improvements, influencing liver processing of fatty acids from other sources. At the end of the intervention when excess IHTG was ameliorated, individual differences in lipogenesis levels remained the driver of residual IHTG. Because DNL is highly responsive to reductions in energy intake, this pathway represents the most highly modifiable biochemical contributor to MASLD. Reductions in hepatic lipid synthesis are currently a target for pharmaceutical development in this field. In the meantime, efforts to help patients lose weight should be more strongly emphasized as a strategy for treatment.

## Methods

### Sex as a biological variable.

Our study included men and women. We analyzed the data for the primary outcomes (changes in body weight, liver fat, fatty acid sources) by sex, and results were not different between men and women (data not shown), and so results are presented as a group.

### Subjects.

The methods have largely been published previously (11, 59, 60) and are described briefly here and in Supplemental Methods. Sixteen non-diabetic, non-smoking Hispanic or African American (self-described) overweight/obese subjects with stable body weight were recruited from local community health fairs and physician referral using a 2-part screening protocol and specific criteria to increase the likelihood of finding subjects with MASLD (Supplemental Figure 1 and Supplemental Methods). Subjects were stratified at baseline into either low (LowLF) or high (HighLF) liver fat groups based on hepatic TG content of <5.6% or ≥5.6%, respectively, as measured by ^1^H-MRS (1). The goal of the present analysis was to determine how weight loss occurring through energy restriction impacted hepatic fatty acid synthesis, TG assembly, and peripheral metabolism in these subjects. Of the 16 subjects who participated in the intervention, all 16 completed the intervention, but 1 subject in the HighLF group was excluded from analysis because of low adherence (missing appointments).

### Study design.

As shown in Figure 1A, this investigation comprised 3 stages: (a) baseline studies, (b) a weight loss intervention period of approximately 5–6 months, and (c) follow-up studies after weight stability was achieved (conducted at approximately the 6-month mark). Baseline and follow-up studies occurred over 3 weeks of testing and consisted of 2 inpatient overnight admissions in the University of Texas Southwestern Medical Center Clinical and Translational Research Center. The first (admission 1) was designed to measure insulin sensitivity using a frequently sampled, insulin-modified, intravenous glucose tolerance test (60), and the second (admission 2) was designed to measure fatty acid metabolism (Figure 1B). Subjects maintained physical activity levels during the testing period, and all foods and beverages were provided to the subjects before and during both admissions based on their habitual dietary patterns (described below).

### Procedures.

Procedures performed at baseline and follow-up were identical and are described in Figure 1B. Prior to admission 1, subjects completed a 3-day food intake diary, 24-hour dietary recall, and an in-depth interview with a registered dietitian to determine usual dietary intake. Food records were analyzed using the Nutrition Data System for Research (NDSR 2009; Minneapolis, Minnesota, USA), and using this information, a weight-maintaining menu was formulated by the dietitian to resemble the subjects’ usual food intake. Food was prepared by the Clinical and Translational Research Center kitchen and delivered to the subject to consume at home for 10 days (3 days before admission 1 and 7 days before admission 2). Alcohol consumption was prohibited from 3 days before admission 1 and throughout the period through admission 2. Subjects had stable body weight and maintained pre-enrollment physical activity levels before and during the 2-week testing period.

On the day of the i.v. glucose tolerance test for assessment of glucose and insulin responses and insulin resistance (Supplemental Methods and ref. 60), body composition measurements were also made by a DEXA scanner (Hologic Discovery W, QDR series), and IHTG measurements by ^1^H-MRS.

The TG–fatty acid composition and sources contributing to plasma VLDL-TG have been shown to reflect liver-TG (12, 14, 61), and therefore the characteristics of VLDL-TG are used to assess liver-TG fluxes. The multiple-stable-isotope procedure used to trace all sources of fatty acids in VLDL-TG palmitate from DNL, meal fat, and plasma FFAs is described in Supplemental Methods, including loading and maintenance doses of deuterium-labeled water (D_2_O), the evening meal containing [U-^13^C_16_]palmitate, and i.v. infusion starting at midnight containing [1,2,3,4-^13^C_4_]palmitate. At noon on day 2, a meal of identical composition and energy to the evening meal was fed. Respiratory gas analysis was conducted via indirect calorimetry (Vmax Encore metabolic cart, Viasys Healthcare) at 0830 hours on day 2 in the fasted state and then again after lunch at 1430 hours to assess the metabolic response to eating. Energy expenditure and fat and glucose oxidation were calculated using standard equations (62); protein catabolism was calculated based on the subject’s controlled protein intake from the previous day (calculated from NDSR dietary analysis) (11).

### Dietary intervention.

The primary goal of the intervention was to achieve weight loss through reduction in energy intake and modification of food quality and composition. The comprehensive dietary intervention methods are further described in Supplemental Methods.

### Outcomes.

Primary outcome measurements included changes induced by weight loss in liver fat, utilization of sources of fatty acids for hepatic lipid palmitate production, and lipoprotein kinetics reflecting hepatic handling of lipids. Secondary outcomes included changes in body weight and composition and metabolic responses in the fasted and fed states (including insulin sensitivity and FFA kinetics). Additional data from ancillary outcomes (e.g., changes in waist circumference, LDL-cholesterol, HDL-cholesterol) are included in Supplemental Table 1.

### Laboratory analyses.

From 1800 on day 1 through 1800 on day 2 of admission 2, blood was collected at 34 time points and plasma immediately separated for measurement of FFA, TG, glucose, and insulin concentrations (Supplemental Methods and ref. 24). For analysis of change with weight loss, fasting values were defined by averaging data from a combination of screening blood draws, those taken just before the intravenous glucose tolerance test, and from admission 2 time points 0600 to 0800. For 1 subject at the follow-up testing period, the fasting insulin value during the i.v. glucose tolerance test (admission 1) was more than 2 SD above the average, but the fasting insulin values during the meal test (admission 2) fell within the SD of all subjects and in line with other metabolic indicators that improved with weight loss (i.e., significant reduction in liver fat, DNL, aspartate transaminase, and alanine transaminase); therefore, for this subject the fasting insulin obtained during the meal test was the only fasting insulin value used at follow-up.

Total TG-rich lipoproteins (TRLs) were isolated from plasma at 22 time points (from midnight until noon, and then also following the lunch meal; described in Supplemental Methods). At midnight, TRL particles contain a mixture of chylomicrons (from the previous evening meal) and hepatically derived VLDL particles (63). With progressive fasting, chylomicrons are cleared from the plasma such that TRL isolated 18 hours after the last meal contains very low concentrations of chylomicrons. Accordingly, this fraction isolated at and after midnight is referred to herein as TRL (containing both chylomicrons and VLDL), whereas after an extended fast (18 hours after the last meal), this fraction is referred to as VLDL (33, 64). An average of the fasting time points (before the lunch meal) was considered to reflect the fasting fatty acid sources for VLDL-TG palmitate. Data for fatty acid sources after the lunch meal were included for the reader’s interest, but because it is not a steady-state period, these data were not assessed statistically. TGs separated from TRL and TG–fatty acids, as well as FFAs from plasma, were prepared for gas chromatography–mass spectrometry (Supplemental Methods and refs. 23, 24, 65–66).

### Quantification of lipid metabolism.

The potential fatty acid sources that contribute to lipoprotein-TG palmitate production (dietary fat, adipose fatty acids, and hepatic DNL) were each labeled with a different palmitate isotope using an established multiple-stable-isotope procedure (23, 24, 33, 65, 66). Details on the multiple-stable-isotope technique are described in Supplemental Methods and ref. 67. Six strategies were used to increase rigor of the isotope labeling methods, including corrections for the calculation of RaFFA for (a) isotopic purity, (b) chylomicron spillover (24), (c) unlabeled fatty acid carried on infused albumin, and (d) the relative contribution of palmitate to plasma FFA composition, and for calculations for VLDL-TG palmitate synthesis, (e) the quantity of lipogenic fatty acids in the plasma FFA pool, and (f) dietary fatty acids in the plasma FFA pool that could recycle into VLDL.

### Statistics.

Calculations were performed using Excel (version 2007, Microsoft). Changes from baseline to follow-up due to weight loss in the HighLF and LowLF groups were compared with 2-way repeated-measures ANOVA (RM-ANOVA) for singular outcomes to assess effects of (a) weight loss and (b) liver fat group (using Statview for Windows v5.0.1, SAS Institute; or GraphPad Prism v10). For time course outcomes during the inpatient metabolic tests, we used 3-way RM-ANOVA (or mixed-effects model if missing values) to assess effects of (a) time over the inpatient testing period, (b) weight loss, and (c) liver fat group (GraphPad). Paired or unpaired 2-tailed *t* tests were performed by Excel (versions 2007 and 365, Microsoft). Analyses with multiple comparisons included Šidák’s (for 2-way RM-ANOVA) or Tukey’s (for 3-way RM-ANOVA) corrections. Relationships between variables were determined by Pearson’s correlation (GraphPad or Excel). Stepwise regression was used to identify which baseline variables best predicted change in liver fat with weight loss; significant variables were entered into a model to assess the predictive power of the model, reported as the adjusted *R*^2^ (SPSS v21, IBM Software). *P* values less than 0.05 were considered statistically significant, with *P* < 0.10 reported as a trend. *P* values were not adjusted for multiple comparisons, but multiple comparisons were limited to the primary outcomes.

### Study approval.

The study was approved by the Institutional Review Board at the University of Texas Southwestern Medical Center (IRB 062007-025), and written informed consent was obtained from all subjects.

### Data availability.

Raw values for data presented in figures and tables are provided in the Supporting Data Values file. Additional data are available upon request.

## Author contributions

JEL collected data, processed and analyzed samples, performed data and statistical analysis, and wrote the manuscript. MARR collected data, analyzed samples, performed data analysis, and provided input into the manuscript. MJV conducted the study, led nutrition counseling, and collected and analyzed data. JDB provided medical and safety management, collected samples, and edited and wrote the manuscript. TR collected and analyzed samples. EJP designed and directed the entire study, collected and analyzed samples, performed data analysis, and wrote the manuscript.

## Supplementary Material

Supplemental data

ICMJE disclosure forms

Supporting data values

## Figures and Tables

**Table 1:**
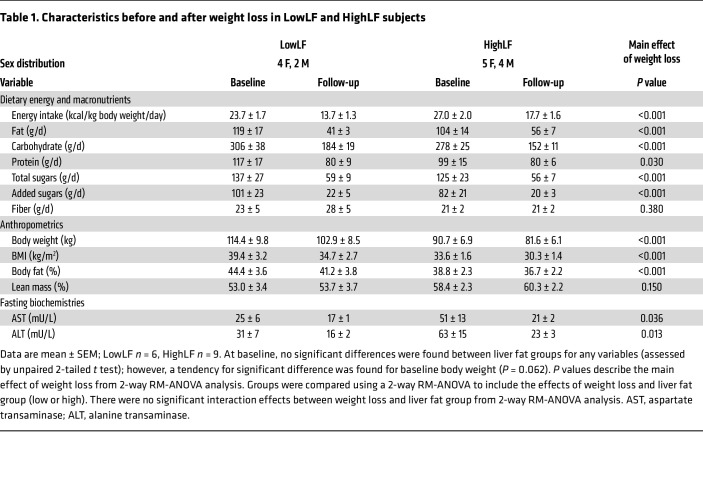
Characteristics before and after weight loss in LowLF and HighLF subjects

**Table 2:**
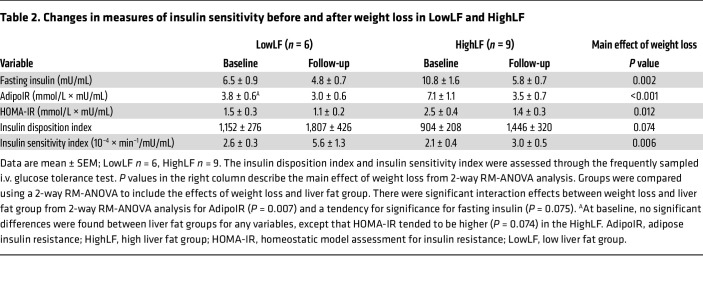
Changes in measures of insulin sensitivity before and after weight loss in LowLF and HighLF

## Version 1 05/01/2025

Electronic publication
