# Supplementary material for: Weight loss in MASLD restores the balance of liver fatty acid sources
Source: J Clin Invest. 2025 May 1;135(9):e174233. doi: 10.1172/JCI174233 (PMC12043097; doi:10.1172/JCI174233)
Supplement: Supplemental data [file jci-135-174233-s059.pdf]

“Weight loss in MASLD restores the balance of liver fatty acid sources”

Jennifer E. Lambert, Maria A. Ramos-Roman, Maressa J. Valdez, Jeffrey D. Browning, Thomas Rogers, Elizabeth J. Parks

Abbreviations used in this document: AdipoIR, adipose insulin resistance; CTRC, Clinical and Translational Research Center; DI, disposition index; DNL, de novo lipogenesis; FFA, free (nonesterified) fatty acids; HighLF, high liver fat participants; LowLF, low liver fat participants; HOMA-IR, homeostatic model assessment for insulin resistance; FSIVGTT, frequently-sampled, intravenous glucose tolerance test; MRS, magnetic resonance spectroscopy; MASLD, metabolic dysfunction-associated steatotic liver disease; RM-ANOVA, repeated measures ANOVA; RaFFA, rate of release of free fatty acids; SI, insulin sensitivity index; TG, triacylglycerols; TRL, TG-rich lipoproteins, VLDL, very low-density lipoproteins.

SUPPLEMENTAL METHODS

Subjects

Many of the methods used in this project have been presented in the previous paper reporting baseline information in these subjects (1). Initial screening criteria included Hispanic or African American adults age 20-64 years, fasting glucose ≤125 mg/dL and/or HbA1c <6.5%, and characteristics of metabolic syndrome (2) including elevated plasma TG and waist circumference, and low HDL-cholesterol, as well as elevated liver enzymes; these criteria were chosen to increase the likelihood of finding subjects with MASLD. Based on the initial screen, eligible subjects (with metabolic syndrome and/or ALT >30, AST >30) were invited to attend a more comprehensive screening visit to rule out diabetes and liver disease from other known causes (including hepatitis, cirrhosis, biliary atresia, cholestasis, and genetic disorders), to obtain a medical and weight history (loss/gain), and measure intrahepatic-TG (IHTG) by 3.0 Tesla ^{1}H-MRS (3, 4). Subjects were excluded if they smoked, had known metabolic abnormalities including elevated thyroid hormone levels, elevated alcohol consumption of >140 g/wk for men and >70 g/wk for women, or were taking medications known to alter metabolism or body weight, including oral hypoglycemic agents, glucocorticoids, thiazide diuretics, valproic acid, beta-blockers, or blood thinners which would preclude blood donations for research purposes. In addition, subjects with auto-immune diseases affecting liver metabolism (e.g., positive alpha-1-antitrypsin test), unusual eating habits (very low-carbohydrate or very high fat intake >45% of energy) or dietary restrictions, and intake of greater

than five dietary supplements (including fish oil) were excluded. Subject recruitment and testing took place from 2008 to 2013.

Procedures

Admission #1 included an insulin-modified, frequently-sampled, intravenous glucose tolerance test (FSIVGTT) performed after a 12-hour overnight fast, as described previously (5). Samples were analyzed for glucose immediately with a bedside analyzer by YSI 2300 Stat Plus (Yellow Springs, Ohio) and insulin within 4 days by ELISA (Millipore, #EZHI-14L). Glucose and insulin responses were analyzed using the minimal model technique and MINMOD Millenium software (6). The homeostatic model assessment for insulin resistance (HOMA-IR) was calculated from fasting insulin and glucose concentrations (described in main text Methods as averages of a combination of screening, IVGTT, and Admission #2 0600 - 0800 timepoints) using the following equation (7):

$$\text{HOMA-IR}=\frac{\text{glucose}\left(\frac{\text{mg}}{\text{dL}}\right)\text{x insulin }(\mu\text{U/mL})}{405}$$

A marker of adipose insulin resistance (AdipoIR) was calculated by multiplying the fasting plasma insulin and FFA concentrations (8).

The last day of admission #1, subjects were given three loading doses of deuterium-labeled water (d_2O) to bring body water enrichments to at least 0.6%. This was followed by three days of consuming 50g doses of 70% deuterated water (d_2O; Cambridge Isotope Laboratories, Inc.; Andover, MA) twice per day (BID) for 3 days, and then once per day for the final 7 days before admission #2 (9).

For admission #2, subjects were admitted to the Clinical Translational Research Center (CTRC) and IV lines were placed. At 1800, the subjects consumed a standardized evening meal (Figure 1B) consisting of cereal, banana, and skim milk, and a cocoa-flavored drink (cocoa, corn oil, heavy cream, sucrose, and skim milk) containing 1g of [U-$^{13}C_{16}$]-palmitate (Isotec/Sigma, Miamisburg, OH) to trace meal fat incorporation into lipoprotein-TG (10). This meal provided 38% of total daily energy needs (50% of

energy from fat, 43% from carbohydrate, and 7% from protein). The fat in the meal was standardized in the liquid drink as a set amount (40g), with balanced proportion of fatty acids (saturated fatty acids 36.2 wt% of the total TG-fatty acids, monounsaturated 28.9%, and polyunsaturated fatty acids 31.9%); the balance of energy in the meal was made up by the cereal, banana, and milk. The subject then fasted for 18 hours, consuming only water or non-caloric, non-caffeine containing beverages after the evening meal until noon the next day, to allow for complete turnover of the plasma VLDL-TG pool. At midnight an IV infusion of [1,2,3,4 $^{13}C_4$]-palmitate (7 µg/kg/min; Isotec; St. Louis, MO) was initiated to measure the contribution of plasma FFA to hepatic-TG synthesis (11) and the rate of adipose- fatty acid flux (see below). During waking time, the subject rested, read, or watched television. Subjects received honoraria to participate in this project. They received $150 for screening and baseline procedures and $300 for follow-up procedures.

Dietary Intervention

The intervention diet was designed to achieve a weight-loss of 1-3 lb/week, corresponding to an energy deficit of ~500 kcal/d compared to the subject's pre-study diet, and caloric quantity was based on anthropometrics and indirect calorimetry analysis. Anthropometrics were used to determine protein requirement for each subject (a minimum of 0.8 g/kg body weight). The main goals of the intervention program were to reduce overall energy intake and modify consumption to improve dietary quality. Either a "low-carbohydrate" diet, based on a goal carbohydrate intake of <200 g/day (~35% of energy) (12), or a "low-fat" diet, based on a goal intake of <28% of energy from fat, as per the AHA Step II recommendations (13), was prescribed to guide dietary macronutrient composition. At the end of 6 months of counseling, both groups consumed similar diets low in processed sugars.

Over the six months, there were eight in-person visits with the Research Dietitian, lasting for 1-2 hours each time. The overall goals were the same for all subjects and included reducing intake of total energy, added sugars, sodium, and processed food, and maintaining adequate protein intake; as such, the nutrition education and behavior counseling was the same for all subjects. During the first portion of

the intervention phase (visits 1-3), subjects were provided with set 7-day meal plans designed by the Research Dietitian. Meal plans were personalized for each subject, taking into consideration lifestyle and food preferences. Energy content and macronutrient composition of each diet was confirmed using NDSR. Along with dietary guidance, subjects were provided with nutrition education including identifying primary food sources of fat, carbohydrate, and protein, reading nutrition labels, and sources of empty calories. The second phase of the intervention (visits 4-6) focused on behavior modification and achieving dietary autonomy. Specific counseling included planning and preparing meals in the home, portion control, and choosing foods when dining out, and counseling was tailored to each individual. For example, if at baseline an individual scored highly on questionnaires that assessed food craving and behavior (e.g. questionnaires TFEQ, FCQ-S and FCQ-T (14, 15), then strategies to identify and prevent emotional eating were the focus of a counseling session. The last portion of the intervention phase (visits 7 and 8) focused on maintenance of dietary behaviors, and subjects were entirely responsible for planning their own meals.

In addition to the in-person visits, compliance to the dietary regimen was assessed by weekly phone calls, measurement of body weight during the in-person visits as well as in the subject's home (on scales calibrated to the research office scale), and three blood draws over the duration of the intervention to monitor standard biochemistries and health indices. Subjects also completed 3-day food records on two occasions during the intervention (typically once during visits 4-6 and again during visits 7-8) to assess dietary compliance, and ensure they were consuming sufficient energy and protein. Body weight and waist:hip ratio was measured at all visits. The waist measurement was determined at the umbilicus while the hip was measured at the largest circumference including the gluteal region.

Laboratory Analyses

Plasma samples collected for measurement of FFA, TG, glucose, and insulin were immediately separated and kept on ice; a preservation cocktail of phenylmethylsulfonyl fluoride, chloramphenicol, gentamicin sulfate, benzamidine and Trolox was added (10). Plasma FFA, TG, and glucose

concentrations were measured enzymatically (kit #991-34891, #461-08992/461-09092, and #439-90901, respectively, Wako Diagnostics, Richmond, VA), and insulin by ELISA (#EZHI-14L, Millipore Corporation, Billerica, MA). PNPLA3 genotype was measured only 10 of the 16 subjects due to technical reasons; genotyping was performed in the lab of Dr. Helen Hobbs at UT Southwestern Medical Center. Of the 10 subjects that were genotyped (5 LowLF subjects and 5 HighLF subjects), 7 were homozygous for the CC variant, 3 were heterozygous (CG), and none were homozygous for the GG variant. Of the 3 that were CG, all were in the HighLF group.

Total TG-rich lipoproteins (TRL) were isolated from plasma by fixed-angle ultracentrifugation at 40,000 RPM for 20 h in a 50.3Ti rotor (Beckman Instruments; Palo Alto, CA) at 15°C (11) at 14 timepoints (from midnight until noon). VLDL particles were obtained by tube slicing to remove the top 2 mL (16), and the bottom 4 mL were kept for isolation of plasma FFA. To prepare FFA for analysis, the 4 mL infranatant that remained after VLDL isolation was extracted with heptane:isopropanol (30:70 mixture; 4 mL) and 1 mol H_2SO_4/L (100 µL). FFA were isolated using thin-layer chromatography with a 80:20:1 hexane:ethyl ether:acetic acid solvent system and silica G plates (20 × 20 cm, 250 μm; Analtech Inc.), derivatized to fatty acid methyl-esters for GC analysis using the method of Lepage and Roy (17). The different species of fatty acids in lipoprotein-TG and plasma FFA were measured by GC with flame ionization detector (18). Two of the 15 subjects' TG samples were not available for TG fatty acid compositional analysis. The group average of 30.4% palmitate was used for these two subjects' calculations. This value was comparable to the value of 30.3±3.7% (SD) that has been found in the literature (unpublished data from our past studies (19) and others (20–26)). TG separated from TRL and TG-fatty acids, as well as FFA from plasma, were prepared for GC/MS as previously described (10, 27). The labeling patterns of TG and FFA were determined by measurement of isotopic enrichments on an Agilent 6890N GC coupled to a 5975 MS detector and 7683B injector (Agilent Technologies, Palo Alto, CA) (28). Palmitate enrichments were determined by selective ion monitoring for m/z of 270, 271, 272, 274, and 286 under electrical ionization using 4-6 point standard curves.

Multiple Stable Isotope Techniques for Quantitation of Lipid Metabolism

Using an established multiple stable-isotope procedure (10, 11, 27–29), the potential fatty acid sources that contribute to lipoprotein-TG palmitate production (dietary fat, adipose fatty acids, and hepatic DNL) were each labeled with a different palmitate isotope, and expressed as both a proportion of total palmitate to reflect intracellular-TG synthesis and the absolute concentration of VLDL-TG palmitate (29). Palmitate serves as the marker for all fatty acids because it is the primary product of DNL, and it is the second most prominent fatty acid in the diet. The contributions of the fatty acid sources were calculated first as a proportion of TRL-TG palmitate, reflecting the flux of fatty acids into intracellular-TG synthesis (11, 29) as demonstrated in liver biopsy studies previously published by us and others (29–31). The proportions of palmitate derived from each source are then multiplied by the absolute concentrations of TRL-TG palmitate to determine the quantitative contributions of these sources to TG-palmitate concentrations (32, 33). It should be noted that even with an extended labeling period there will likely be a proportion of VLDL-TG-palmitate that remains unlabeled due to the use of visceral or intrahepatic stores of TG-palmitate (29, 34, 35). Lastly, fractional lipoprotein-TG turnover rates were calculated by modeling the rise to plateau enrichment of [$^{13}C_4$]-palmitate into VLDL-TG using SigmaPlot (V12.0; Systat Software Inc., San Jose, CA) (10).

Mass isotopomer distribution analysis was used to calculate the percentage of newly-made palmitate in VLDL (10, 36). DNL was calculated from samples throughout the evening (from midnight to noon). Fasting DNL was defined as the percent contribution of newly-synthesized fatty acid in the VLDL-TG-palmitate pool expressed as an average of the last three fasting blood samples taken. During the d_2O labeling period (10 days), fatty acid sources were determined from different pools. DNL-derived fatty acids in VLDL-TG may arise from newly-synthesized fatty acid in the liver, as well as previously synthesized fatty acids that have been stored in IHTG, DNL-fatty acids that have been exported in VLDL-TG and entered the plasma FFA pool through lipolysis of either VLDL particles. The model does not include adipose-synthesized DNL fatty acids released by lipolysis because the total adipose pool is large and durations of d_2O labeled of much longer duration (months, compared to our 10 days) is

needed to label up that pool. The proportion of DNL-palmitate in the plasma FFA pool was measured and taken into consideration in the calculation of the proportion of DNL-palmitate in VLDL-TG-palmitate. Nonetheless, we consider that the DNL label in FFA comes from VLDL-DNL, and thus is also thought to originate from the liver. Scientifically, if one is interested in the original sources of fatty acids contributing to VLDL-TG synthesis, then calculations would account for only three sources in VLDL-TG (adipose, diet and DNL). This view is evident in figure 5 and emphasizes that any apparent DNL fatty acids in VLDL-TG arose from liver production, whether or not there is spillover of the label from VLDL back into the plasma FFA pool and recycling of these DNL FFA through the liver back into VLDL. By contrast, if one is focused on the contribution of all sources of plasma FFA to VLDL synthesis, spillover into the FFA pool from the chylomicrons and VLDL can be accounted for (as depicted in the paper's figure 6).

Six strategies were used to increase rigor of the isotope labeling methods including corrections for the calculation of RaFFA for i) isotopic purity, ii) chylomicron spillover (10), iii) unlabeled fatty acid carried on infused albumin, iv) the relative contribution of palmitate to plasma fatty acid composition, and for calculations for VLDL-TG synthesis v) the quantity of lipogenic fatty acids in the plasma FFA pool and vi) dietary fatty acids in the plasma FFA pool that could recycle into VLDL. Specifically, a correction was applied to the calculation of FFA arising from adipose tissue in TRL-TG to account for fatty acids that do not arise from adipose lipolysis (i.e., that instead arise from dietary spillover and VLDL spillover). Thus, the rate of appearance of adipose-fatty acid release (Adipose RaFFA) was calculated using standard dilution equations and corrected for the presence of dietary and DNL spillover (27).

A standard set of assumptions was used for the calculations including serum volume was 4.5% of total body weight and the incorporation of the plasma FFA label into VLDL-TG was used to calculate VLDL-TG production rate and fractional catabolic rate (10). VLDL-TG clearance rate (mL/min) was determined by dividing the production rate (μmol/min) by the fasting VLDL-TG concentration (mmol/L). Fasting RaFFA was determined as the average of measurements taken throughout the morning during

a steady-state period (0500-1145). The IV infusate was measured by GC and GC/MS to determine the fatty acid composition and the amount of unlabeled fatty acids present from the albumin used in the infusate (10). The calculations for RaFFA were adjusted for unlabeled fatty acids occurring in the infusate solution (10). The amount of fatty acids arising from spillover of meal-fat was also determined by the presence of the dietary label ([U-^{13}C]-palmitate) found in the FFA to correct for contribution of these fatty acids in order to determine FFA arising from adipose-TG lipolysis alone (10). The composition of plasma FFA was determined by GC and the percentage of palmitate used to determine total RaFFA (10). The rate of plasma FFA incorporated into VLDL-TG was calculated as a proportion of the available FFA in plasma (i.e. production rate of VLDL-TG-FFA multiplied by 3 to reflect three fatty acids per TG and divided by RaFFA) as per Fabbrini et al. (35), with an additional correction applied to reflect FFA incorporation into VLDL-TG as a proportion of the plasma FFA taken up by the liver, estimated as 21% by Iozzo et al. (37):

$$\text{Production rate of VLDL-TG from FFA } \left(\frac{\mu\text{mol}}{\text{min}}\right) = \left(\text{VLDL-TG production rate } \frac{\mu\text{mol}}{\text{min}}\right) \text{ x VLDL-TG-palmitate \%FFA}$$

and then:

$$\text{VLDL-TG-FFA as \% of RaFFA} = \frac{\text{Production rate of VLDL-TG from FFA } \left(\frac{\mu\text{mol}}{\text{min}}\right) \text{x 3 FA per TG}}{\text{Adipose RaFFA } \left(\frac{\mu\text{mol}}{\text{min}}\right) \text{x 21\%}}$$

For this measurement, there was n=5 for LowLF due to missing VLDL kinetic data at follow-up.

Sample size estimate

Statistical power calculations were based on estimates of variability (38–40). In measurements of liver lipid flux, inter-individual variability can be much less than treatment differences when robust effects are at work (41). Since the hypotheses are based on metabolic changes associated with body weight loss, a goal sample size of 20 subjects was derived from the expected changes in body weight and liver fat (38–40). For the present study, each subject was to serve as his/her own control and a meaningful reduction in body weight was designated as 6% at the end of the weight-loss phase and during weight stabilization. This amount of loss was possible over the 6-month period of the study given our (42), and

other (43, 44) experiences working with this population. Using a power calculation with Z_a of 1.96 and Z_b for 80 and 90%, if the true change in weight is 6% or greater (with a standard deviation for that change of 4%), we expected to have an 80% chance of detecting a significant change in liver fat with an n=16, and a 90% chance of detecting it with 20 subjects. The final weight loss experienced in this study was 10±2%.

REFERENCES

1. Lambert JE, et al. Increased de novo lipogenesis is a distinct characteristic of individuals with nonalcoholic fatty liver disease. *Gastroenterology.* 2014;146(3):726–735.

2. Grundy SM, et al. Diagnosis and management of the metabolic syndrome: an American Heart Association/National Heart, Lung, and Blood Institute scientific statement. *Curr Opin Cardiol.* 2006;21(1):1–6.

3. Szczepaniak LS, et al. Magnetic resonance spectroscopy to measure hepatic triglyceride content: prevalence of hepatic steatosis in the general population. *Am J Physiol Endocrinol Metab.* 2005;288(2):E462–E468.

4. Browning JD, et al. Prevalence of hepatic steatosis in an urban population in the United States: impact of ethnicity. *Hepatology.* 2004;40(6):1387–1395.

5. Ramos-Roman MA, et al. Insulin activation of plasma nonesterified fatty acid uptake in metabolic syndrome. *Arterioscler Thromb Vasc Biol.* 2012;32(8):1799–1808.

6. Boston RC, et al. MINMOD Millennium: a computer program to calculate glucose effectiveness and insulin sensitivity from the frequently sampled intravenous glucose tolerance test. *Diabetes Technol Ther.* 2003;5(6):1003–1015.

7. Matthews DR, et al. Homeostasis model assessment: insulin resistance and beta-cell function from fasting plasma glucose and insulin concentrations in man. *Diabetologia.* 1985;28(7):412–419.

8. Søndergaard E, et al. How to measure adipose tissue insulin sensitivity. *J Clin Endocrinol Metab.* 2017;102(4):1193–1199.

9. Turner SM, et al. Measurement of TG synthesis and turnover in vivo by 2H2O incorporation into the glycerol moiety and application of MIDA. *Am J Physiol Endocrinol Metab.* 2003;285(4):E790–E803.

10. Barrows BR, Parks EJ. Contributions of different fatty acid sources to very low-density lipoprotein-triacylglycerol in the fasted and fed states. *J Clin Endocrinol Metab.* 2006;91(4):1446–1452.

11. Parks EJ, et al. Effects of a low-fat, high-carbohydrate diet on VLDL-triglyceride assembly, production, and clearance. *J Clin Invest.* 1999;104(8):1087–1096.

12. Westman EC, et al. Low-carbohydrate nutrition and metabolism. *Am J Clin Nutr.* 2007;86(2):276–284.

13. Krauss RM, et al. AHA Dietary Guidelines: revision 2000: A statement for healthcare professionals from the Nutrition Committee of the American Heart Association. *Circulation.* 2000;102(18):2284–2299.

14. Cepeda-Benito A, et al. The development and validation of Spanish versions of the State and Trait Food Cravings Questionnaires. *Behav Res Ther.* 2000;38(11):1125–1138.

15. Stunkard AJ, Messick S. The three-factor eating questionnaire to measure dietary restraint, disinhibition and hunger. *J Psychosom Res.* 1985;29(1):71–83.

16. Lee JJ, et al. Palmitoleic acid is elevated in fatty liver disease and reflects hepatic lipogenesis. *Am J*

Clin Nutr. 2015;101(1):34–43.
17. Lepage G, Roy CC. Specific methylation of plasma nonesterified fatty acids in a one-step reaction. *J Lipid Res.* 1988;29(2):227–235.
18. Chavez-Jauregui RN, et al. Dynamics of fat absorption and effect of sham feeding on postprandial lipema. *Gastroenterology.* 2010;139(5):1538–1548.
19. Syed-Abdul MM, et al. Fatty acid synthase inhibitor TVB-2640 reduces hepatic de novo lipogenesis in males with metabolic abnormalities. *Hepatology.* 2020;72(1):103–118.
20. Ruiz-Gutiérrez V, et al. Composition of human VLDL triacylglycerols after ingestion of olive oil and high oleic sunflower oil. *J Nutr.* 1998;128(3):570–576.
21. Perona JS, et al. Reduction in systemic and VLDL triacylglycerol concentration after a 3-month Mediterranean-style diet in high-cardiovascular-risk subjects. *J Nutr Biochem.* 2010;21(9):892–898.
22. Jensen RG, et al. Cholesteryl ester and triacylglycerol fatty acids in type V hyperlipidemia. *Lipids.* 1979;14(7):691–694.
23. Clandinin MT, et al. Increasing the dietary polyunsaturated fat content alters whole-body utilization of 16:0 and 10:0. *Am J Clin Nutr.* 1995;61(5):1052–1057.
24. Aarsland A, Wolfe RR. Hepatic secretion of VLDL fatty acids during stimulated lipogenesis in men. *J Lipid Res.* 1998;39(6):1280–1286.
25. Layne KS, et al. Normal subjects consuming physiological levels of 18:3(n-3) and 20:5(n-3) from flaxseed or fish oils have characteristic differences in plasma lipid and lipoprotein fatty acid levels. *J Nutr.* 1996;126(9):2130–2140.
26. The LipidWeb by LipidMaps. Plasma Lipoproteins - Composition and Structure. https://www.lipidmaps.org/resources/lipidweb/lipidweb_html/lipids/simple/lipoprot/index.htm. Updated July 10, 2024. Accessed July 13, 2024
27. Barrows BR, et al. Spillover of dietary fatty acids and use of serum nonesterified fatty acids for the synthesis of VLDL-triacylglycerol under two different feeding regimens. *Diabetes.* 2005;54(9):2668–2673.
28. Timlin MT, et al. Increased dietary substrate delivery alters hepatic fatty acid recycling in healthy men. *Diabetes.* 2005;54(9):2694–2701.
29. Donnelly KL, et al. Sources of fatty acids stored in liver and secreted via lipoproteins in patients with nonalcoholic fatty liver disease. *J Clin Invest.* 2005;115(5):1343–1351.
30. Peter A, et al. Hepatic lipid composition and stearoyl-coenzyme A desaturase 1 mRNA expression can be estimated from plasma VLDL fatty acid ratios. *Clin Chem.* 2009;55(12):2113–2120.
31. Syed-Abdul MM, et al. Isotope labeling and biochemical assessment of liver-TAG in patients with different levels of histologically-graded liver disease. *J Nutr.* 2023;153(12):3418–3429.
32. Santoro N, et al. Hepatic de novo lipogenesis in obese youth is modulated by a common variant in

the GCKR gene. *J Clin Endocrinol Metab.* 2015;100(8):E1125–E1132.
33. Baykal AP, et al. Leptin decreases de novo lipogenesis in patients with lipodystrophy. *JCI Insight.* 2020;5(14):e137180.
34. Parks EJ. Effects of a high-carbohydrate diet on VLDL particle synthesis and composition in normolipidemic and hypertriglyceridemic individuals. *Diabetes.* 1998;47(Suppl 1):A28.
35. Fabbrini E, et al. Alterations in adipose tissue and hepatic lipid kinetics in obese men and women with nonalcoholic fatty liver disease. *Gastroenterology.* 2008;134(2):424–431.
36. Hellerstein MK, Neese RA. Mass isotopomer distribution analysis at eight years: theoretical, analytic, and experimental considerations. *Am J Physiol.* 1999;276(6):E1146–E1170.
37. Iozzo P, et al. Defective liver disposal of free fatty acids in patients with impaired glucose tolerance. *J Clin Endocrinol Metab.* 2004;89(7):3496–3502.
38. Tiikkainen M, et al. Effects of identical weight loss on body composition and features of insulin resistance in obese women with high and low liver fat content. *Diabetes.* 2003;52(3):701–707.
39. Wood RJ, et al. Carbohydrate restriction alters lipoprotein metabolism by modifying VLDL, LDL, and HDL subfraction distribution and size in overweight men. *J Nutr.* 2006;136(2):384–389.
40. Landry N, et al. Whole-body fat oxidation rate and plasma triacylglycerol concentrations in men consuming an ad libitum high-carbohydrate or low-carbohydrate diet. *Am J Clin Nutr.* 2003;77(3):580–586.
41. Hellerstein MK. Synthesis of fat in response to alterations in diet: insights from new stable isotope methodologies. *Lipids.* 1996;31(Suppl):S117–S125.
42. Browning JD, et al. A low-carbohydrate diet rapidly and dramatically reduces intrahepatic triglyceride content. *Hepatology.* 2006;44(2):487–488.
43. Aude YW, et al. The national cholesterol education program diet vs a diet lower in carbohydrates and higher in protein and monounsaturated fat: a randomized trial. *Arch Intern Med.* 2004;164(19):2141–2146.
44. Farnsworth E, et al. Effect of a high-protein, energy-restricted diet on body composition, glycemic control, and lipid concentrations in overweight and obese hyperinsulinemic men and women. *Am J Clin Nutr.* 2003;78(1):31–39.

Supplemental Figure 1. Participant flow diagram depicting original subject recruitment (as previously reported in supplemental reference 1, Lambert, *Gastroenterology* 2014) and through the weight-loss intervention study

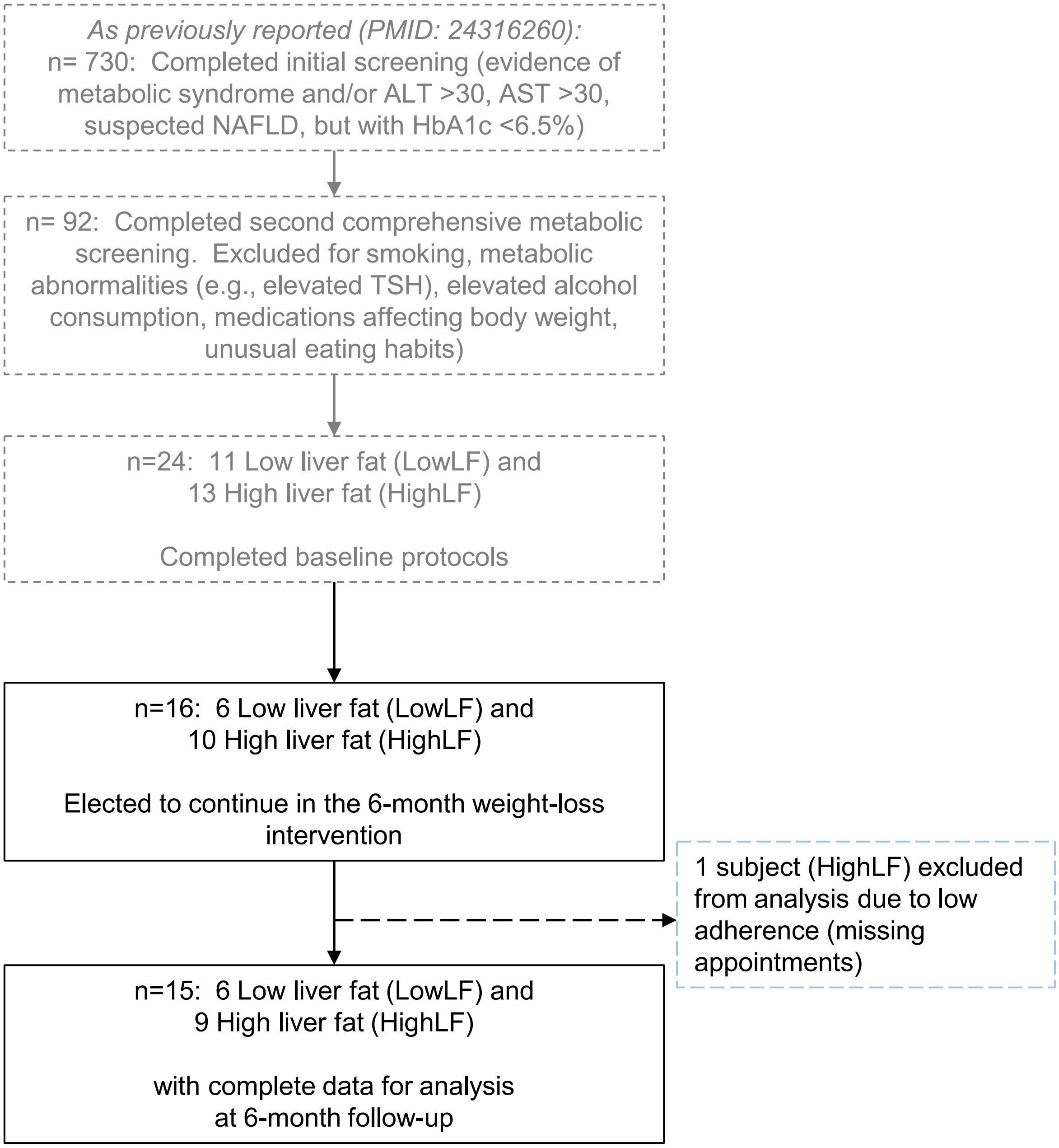

Supplemental Figure 2. Baseline and follow-up liver histology from 4 research subjects.

A

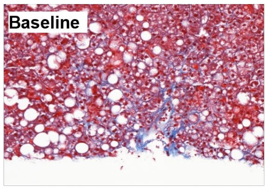

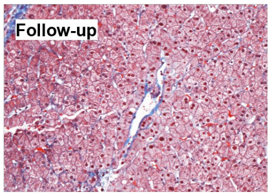

Participant #4, female (33y)

Baseline Histology (Trichrome 200x)
Mild pericellular fibrosis adjacent to central veins. Ballooning degeneration of hepatocytes.

	Baseline	Follow-up
Body weight	68.8 kg	61.5 kg
Plasma ALT	63 U/L	11 U/L
Plasma TG	1.90 mmol/L	0.97 mmol/L
IVGTT-SI	1.84	3.10
Liver fat	27.3%	1.8%
NAFLD Score	1	0

Follow-Up Histology (Trichrome 200x)
Healthy liver demonstrating resolution of steatosis and fibrosis.

B

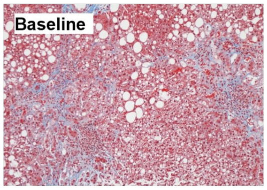

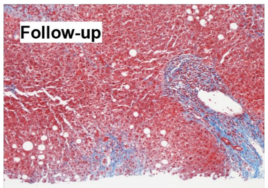

Participant #2, female (46y)

Baseline Histology (Trichrome 100x)
Macro- and microvesicular steatosis with hepatocellular ballooning degeneration. Extensive "chicken wire" pericellular fibrosis around central vein and inflammation.

	Baseline	Follow-up
Body weight	80.5 kg	72.9 kg
Plasma ALT	99 U/L	15 U/L
Plasma TG	1.17 mmol/L	0.97 mmol/L
IVGTT-SI	3.09	2.17
Liver fat	21.3%	7.0%
NAFLD Score	6	2

Follow-up Histology (Trichrome 100x)
Significant reduction in steatosis. Fibrosis graded lower when viewing multiple sections. Inflammation persists, but significantly less hepatocellular degeneration found.

C

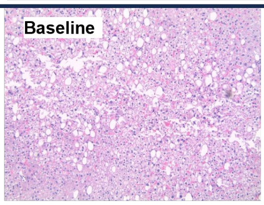

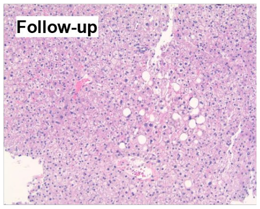

Participant #1, female (48y)

Baseline Histology (H&E 10x)
Steatosis, microvesicular, occupying 15% of liver tissue surface area.

	Baseline	Follow-up
Body weight	59.6 kg	52.9 kg
Plasma ALT	56 U/L	26 U/L
Plasma TG	1.77 mmol/L	1.05 mmol/L
IVGTT-SI	4.75	2.45
Liver fat	19.9%	6.6%
NAFLD Score	1	1

Follow-Up Histology (H&E 10x)
Steatosis, microvesicular, occupying 5% of liver tissue surface area.

D

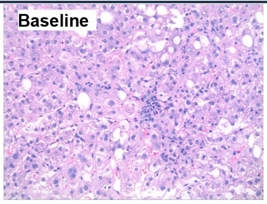

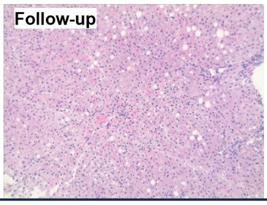

Participant #5, female (46y)

Baseline Histology (H&E 20x)
Steatohepatitis with portal, moderate perivenular, and occasional areas of bridging fibrosis.

	Baseline	Follow-up
Body weight	86.7 kg	79.2 kg
Plasma ALT	166 U/L	34 U/L
Plasma TG	1.04 mmol/L	0.86 mmol/L
IVGTT-SI	1.23	1.06
Liver fat	17.5%	6.2%
NAFLD Score	6	4

Follow-up Histology (H&E 10x)
Similar degree of steatosis to that present in the earlier biopsy, but less parenchymal inflammation and ballooning degeneration of hepatocytes.

This figure shows histology data from four subjects who underwent repeat liver biopsies at baseline and follow-up. Abbreviations: ALT – alanine transaminase; IVGTT-SI = insulin sensitivity index determined from the frequently-sampled IV glucose tolerance test; TG – triglyceride.

Supplemental Figure 3. Fasting plasma glucose, insulin, FFA, and TG levels before and after weight-loss in low liver fat (LowLF) and high liver fat (HighLF) subjects

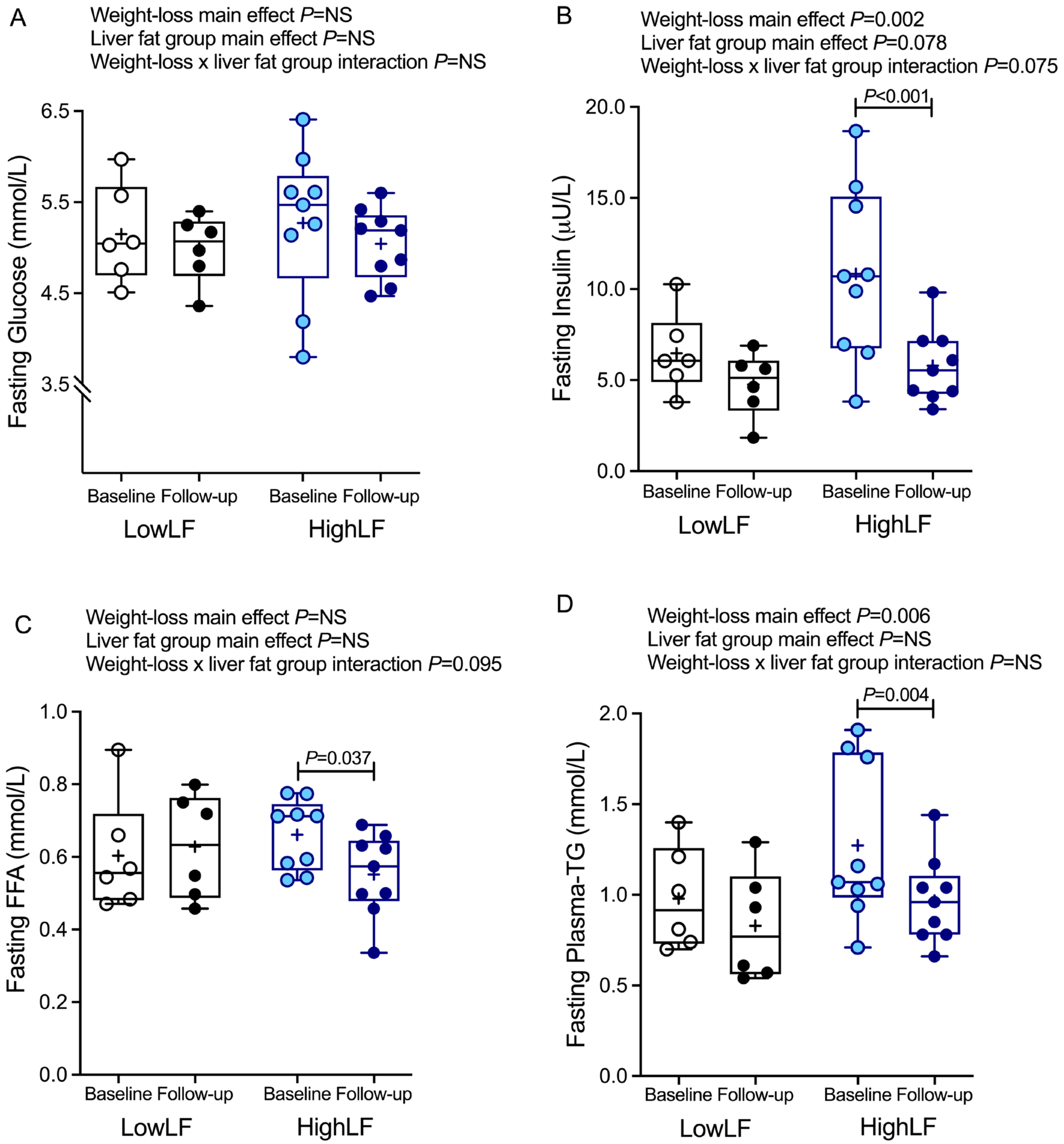

Data are mean ± SEM for LowLF (n=6) and HighLF (n=9) for plasma concentrations of (**A**) glucose, (**B**) insulin, (**C**) free fatty acids, and (**D**) triacylglycerols. Bar graphs represent the average fasting concentrations of metabolites, the mean is indicated by "+" (may be behind a data point), boxes represent the 25th and 75th percentiles, middle line in the box represents the median, and the whiskers the min and max values. *P*-values above the graphs indicate main effects of weight-loss, liver fat group, and weight-loss x liver fat group interaction effects determined by two-way repeated measures ANOVA. *P*-values on the graphs represent sub-group comparisons from the two-way RM-ANOVA in the change from baseline to follow-up within LowLF and HighLF groups.

Supplemental Figure 4. Absolute contributions of fatty acid sources contributing to fasting VLDL-TG palmitate concentrations before and after weight-loss in subjects with LowLF and HighLF

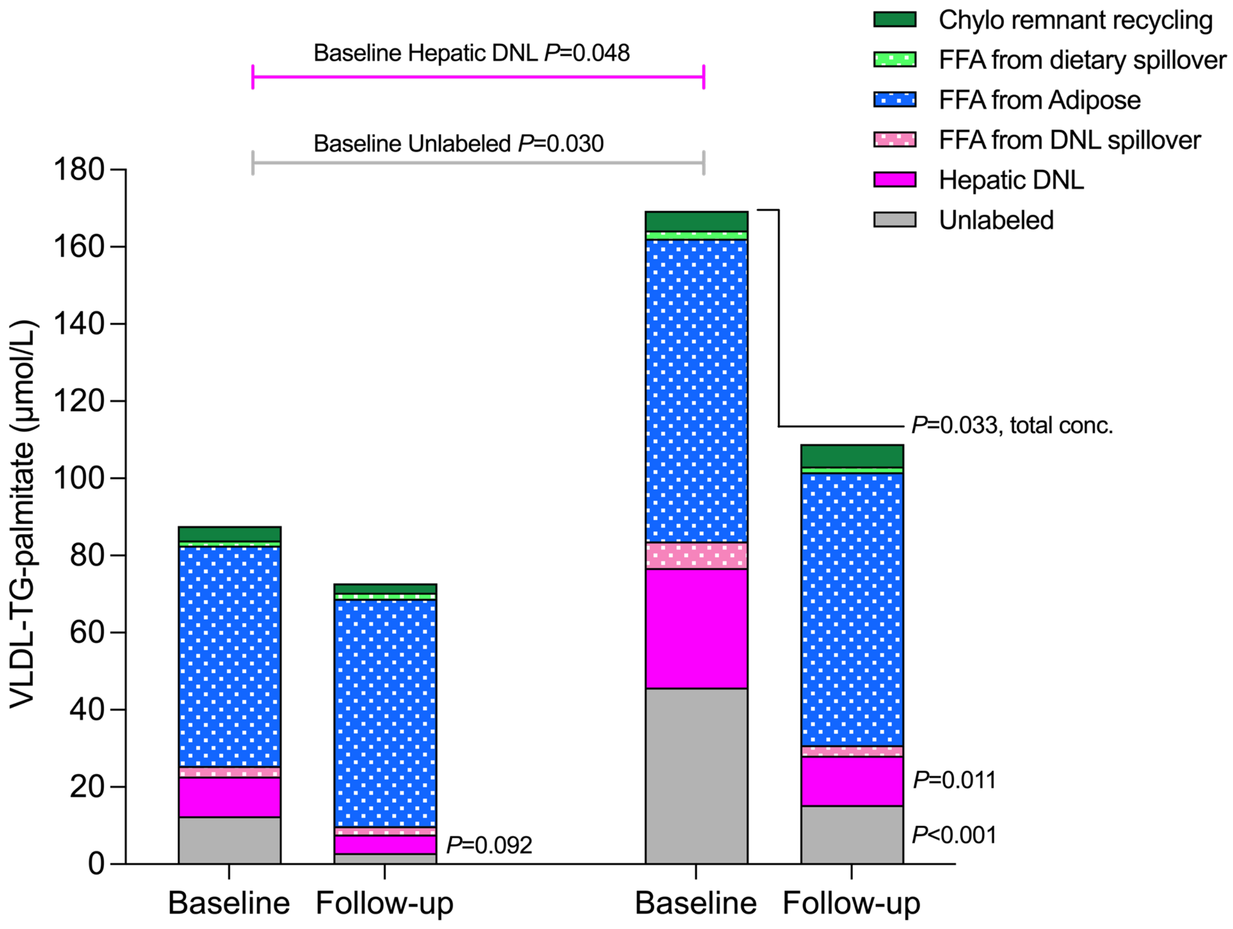

Data are means, with variances removed for clarity for LowLF (n=6) and HighLF (n=9). When the proportional sources of fatty acid found in VLDL-TG palmitate are multiplied by the fasting VLDL-TG palmitate concentration, the absolute contributions of fatty acid sources to VLDL-TG can be calculated. Changes due to weight-loss were assessed between the groups using two-way repeated measures ANOVA. There were main effects of weight-loss (*P*=0.006) and liver fat group (*P*=0.066) for amount of fatty acid from DNL, as well as weight-loss by liver fat group interaction (*P*=0.006) for amount of fatty acid unaccounted for. The *P*-value above the bar graphs highlights differences between the two groups at baseline (assessed by unpaired t-test). *P*-values next to the bars represent baseline vs. follow-up comparisons within a single group (assessed by paired t-test).

Supplemental Figure 5. Relationships between changes in liver fat, and concentrations of VLDL-TG palmitate, unlabeled palmitate, and that labeled from hepatic DNL

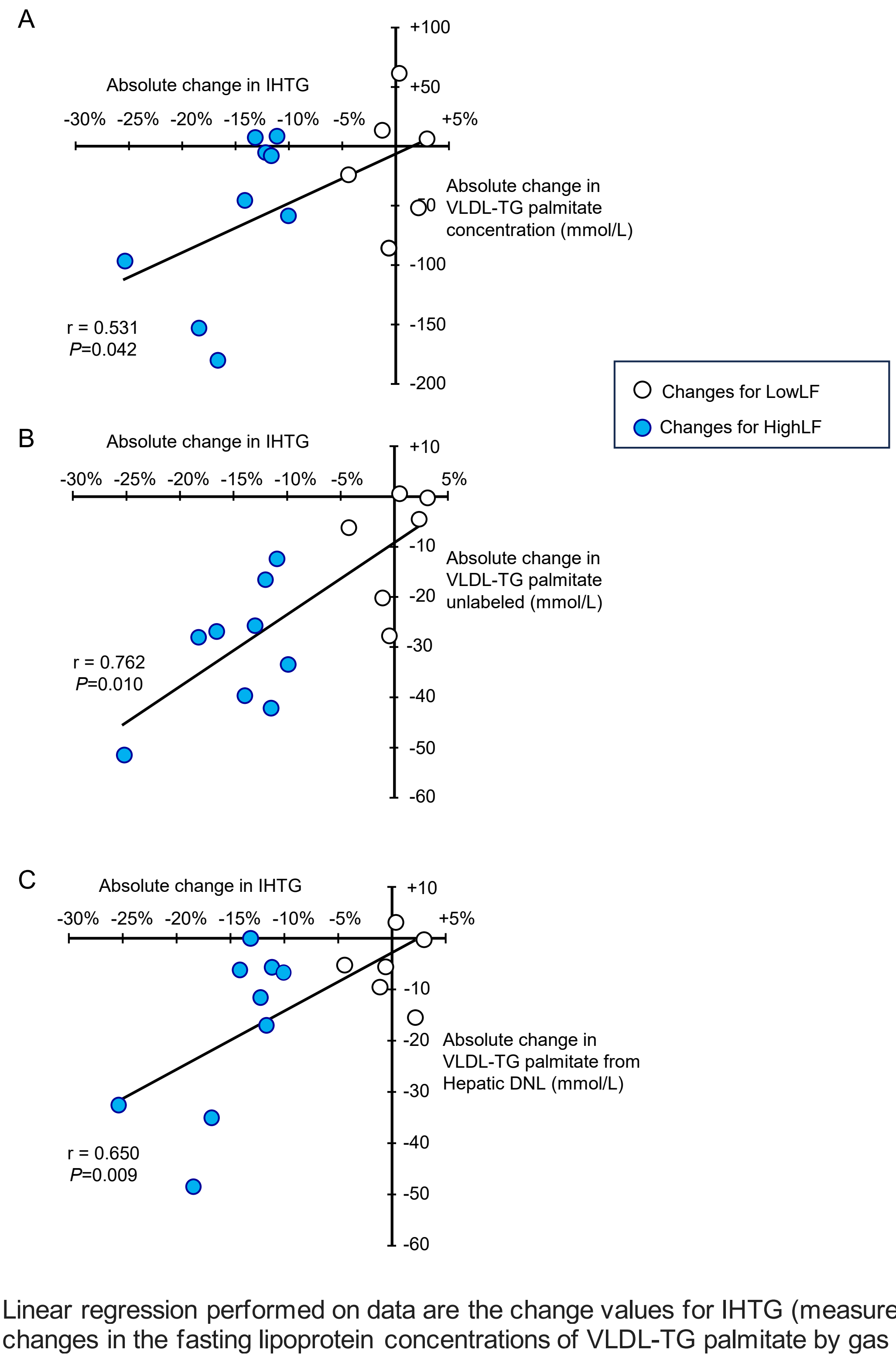

Linear regression performed on data are the change values for IHTG (measured by MRS), and (**A**) changes in the fasting lipoprotein concentrations of VLDL-TG palmitate by gas chromatography, (**B**) the unlabeled portion of palmitate as assessed by mass spectrometry, and (**C**) the presence of newly-made fatty acids in TG-palmitate assessed by mass spectrometry. Of the six sources of VLDL-TG palmitate presented in **supplemental figure 4**, hepatic DNL was the only one that correlated with change in IHTG.

Supplemental Table 1. Additional data from ancillary outcomes

Sex distribution<br>Race distribution<br>**Variable**	**LowLF**<br>**4 F : 2 M**<br>**1 H : 5 AA**<br>**Baseline**	 <br><br>**Follow-up**	**HighLF**<br>**5 F : 4 M**<br>**5 H : 4 AA**<br>**Baseline**	 <br><br>**Follow-up**	**Main effect of weight-loss**<br>***P*-value**
Waist:Hip ratio	0.95 ± 0.03	0.89 ± 0.03	0.97 ± 0.04	0.88 ± 0.02	0.002
Trunk fat (%)	21.8 ± 2.2	20.0 ± 1.9	20.8 ± 1.6	20.2 ± 1.3	0.060
TG (mmol/L)	0.98 ± 0.11	0.83 ± 0.12	1.27 ± 0.14	0.97 ± 0.08	0.006
TC (mmol/L)	4.97 ± 0.15	4.75 ± 0.34	5.09 ± 0.28	4.59 ± 0.17	0.037
LDLc (mmol/L)	3.26 ± 0.07	3.07 ± 0.26	3.23 ± 0.27	2.67 ± 0.17	0.015
HDLc (mmol/L)	1.20 ± 0.12	1.22 ± 0.13	1.26 ± 0.06	1.43 ± 0.07	0.045
IVGTT-AIRg (mU/mL x min)	537 ± 187	536 ± 232	487 ± 106	583 ± 128	0.301
Energy Expenditure (kcal/kg/day) [A]					
Fasting	13.3 ± 1.3	13.5 ± 1.3	15.6 ± 0.9	16.0 ± 1.2	0.588
Fed [B]	15.7 ± 1.4	26.3 ± 0.7	17.9 ± 1.3	32.2 ± 2.2	<0.001

Data are presented as mean ± SEM for subjects with low liver fat (LowLF, 4 females and 2 males; 1 Hispanic and 4 African American) and high liver fat (HighLF, 5 females and 4 males; 5 Hispanic and 4 African American).

Abbreviations: HDLc, high-density lipoprotein cholesterol; IVGTT-AIRg, frequently-sampled, intravenous glucose tolerance test, acute insulin response to glucose; LDLc, low-density lipoprotein cholesterol; TC, plasma total cholesterol; TG, plasma triacylglycerols.

At baseline, no significant differences were found between liver fat groups for any of the variables. Groups were compared using a two-way repeated measures ANOVA to assess the effects of weight-loss and liver fat group (LowLF and HighLF) and interaction effect (weight-loss x liver fat group interaction). *P*-values in the table describe the main effect of weight-loss. No interaction effects for weight-loss x liver fat group were observed.

[A] One LowLF subject at baseline and one HighLF subject at follow-up had missing data (due to equipment malfunction) and therefore LowLF n=5 and HighLF n=8 for energy expenditure measurements.
[B] Main effect of liver fat group x time: *P*=0.050 for fed-state energy expenditure.
